# Investigating Project Care UK, a Web-Based Self-Help Single-Session Intervention for Youth Mental Health: Program Evaluation

**DOI:** 10.2196/72077

**Published:** 2025-06-18

**Authors:** Maria Elizabeth Loades, Grace Perry, Noah Marshall

**Affiliations:** 1 Department of Psychology University of Bath Bath United Kingdom

**Keywords:** depression, adolescent mental health, scalable interventions, digital mental health intervention, early help, single-session intervention

## Abstract

**Background:**

Psychological distress becomes more common during adolescence, yet many young people struggle to access clinic-based mental health care. Digital, self-help single-session interventions (SSIs) could extend current provision and overcome barriers to help seeking.

**Objective:**

This study aims to pilot Project Care UK, a self-compassion–focused SSI, to examine its feasibility, acceptability, and preliminary efficacy for UK adolescents aged between 13 and 18 years.

**Methods:**

We used a single-arm, within-subjects pre-post intervention program evaluation. Consenting participants completed a demographic survey and clinical measures at baseline. Self-assessments of hope, hopelessness, negative beliefs about self-compassion, and help seeking were measured immediately before and after the intervention. Acceptability and feasibility were measured after the intervention using the Program Feedback Scale and study completion metrics. Preliminary efficacy was evaluated using linear mixed-effects models. The study protocol was preregistered on the Open Science Framework before publication.

**Results:**

Of the 813 individuals who gave consent for the study, 714 (87.8%) initiated the preintervention assessment survey, 610 (75%) initiated the intervention, 341 (41.9%) initiated the Program Feedback Scale, and 329 (40.5%) initiated the postintervention assessment survey. The sample consisted of adolescents (mean age 15.38, SD 1.58 y) who were predominantly assigned female sex at birth, were White, and were nonheterosexual. Intervention completers widely endorsed the intervention as acceptable. Significant, favorable pre- and postintervention changes were observed across all outcome measures, including increased hope (Cohen *d*=0.72, *P*<.001), decreased hopelessness (Cohen *d*=–0.73, *P*<.001), and reduced negative beliefs about self-compassion (Cohen *d*=–0.64, *P*<.001). No significant changes were observed for help-seeking intentions.

**Conclusions:**

Although not all participants completed the study, our findings show that recruiting adolescents in the United Kingdom is feasible; completers indicated that the intervention was acceptable, and they showed improvements in the proximal outcomes of hope, hopelessness, and beliefs about self-compassion. More extensive follow-up over time and comparator intervention analyses would allow more robust conclusions to be drawn.

## Introduction

### Background

Adolescence is a critical period of marked physical, cognitive, social, and emotional development [1], during which psychological distress (eg, depression and anxiety) becomes increasingly common [2]. However, many adolescents will never access treatments [3,4]. In the United Kingdom, a large-scale school-based survey study found that approximately one-third of adolescents had a perceived unmet need for mental health help [5]. Adolescents from underserved groups (eg, adolescents with disabilities; those identifying as lesbian, gay, bisexual, transgender, queer, intersex, and asexual; and ethnic minority adolescents) are particularly less likely to access treatments [6]. There are many potential barriers to accessing mental health services, such as stigma, long waiting lists, inconvenient appointment times, the requirement of parental permission, and a lack of knowledge about where and how to seek help [7-10]. If untreated, psychological distress can escalate, and short- and long-term functioning can be negatively impacted [11,12]. Furthermore, UK adolescents have emphasized through priority-setting exercises the importance of receiving timely support at the onset of mental health challenges [13,14]. To expand upon current mental health provision in the United Kingdom, this study seeks to evaluate the feasibility, acceptability, and preliminary efficacy of a scalable solution, a web-based self-help self-compassion–focused single-session intervention (SSI) for adolescents in the United Kingdom.

SSIs are intentionally designed as stand-alone encounters, which do not assume or preclude repeat use [15,16] and offer a means of providing evidence-based content at scale. SSIs can be offered in a self-help delivery format, without the need for any therapist or human supporter involvement. Self-help SSIs can be completed at any time (ie, on demand or when the user wants to access them) and, if online, from any device that connects to the internet. Self-help SSIs can be accessed anonymously. Importantly, self-help SSIs can be designed to include the key takeaway messages of therapeutic interventions necessary for change within 1 session [17] and thereby are a means of delivering active ingredients from evidence-based treatments (eg, behavioral activation). For adolescents, digital self-help SSIs overcome many known barriers to accessing treatment, such as stigma, long waiting lists, and inconvenient appointment times [7] while not assuming repeat attendance or use, which have been problematic for other approaches to digital and self-help therapeutic interventions [18,19].

Growing evidence indicates that digital self-help SSIs are feasible, acceptable, safe, and effective in reducing depression symptoms and improving quality of life and other outcomes for adolescents [20]. Several of the best-evaluated SSIs have been developed by the Lab for Scalable Mental Health in the United States. Their suite of open-access SSIs [21] includes Project Care (also known as the Teen Goals Project), which aims to develop self-compassion and reduce self-hate. Two open platform trials in the United States have provided evidence that Project Care appears to be feasible and acceptable to American adolescents, with positive proximal outcomes, including decreases in hopelessness and improvements in perceived agency, which yielded large and medium within-subjects effect sizes (Cohen *d*_z_=1.01 and Cohen *d*_z_=0.65), respectively, immediately after the intervention [22,23]. Project Care has not been evaluated in a randomized controlled trial, but 2 other digital self-help SSIs from the suite have been subject to this level of scrutiny [24]. In comparison to an online activity control condition, both Project Personality (growth mindset–based SSI) and Project ABC (behavior activation–based SSI) were effective in reducing depression symptoms 3 months later in a large sample of individuals aged between 13 and 16 years around the time of the COVID-19 pandemic. Moreover, existing studies found that the uptake of SSIs by adolescents from underserved groups is higher than in traditional clinic-based services [24,25], and existing SSIs appear to be effective across demographic groups [26]. Thus, digital self-help SSIs, such as Project Care, could be a useful addition to existing provisions in the UK context, but they have yet to be extensively implemented and tested. Many questions remain regarding implementation strategies and adaptations to existing SSIs, which may be required for this context.

Despite the existing evidence being strongest for 2 other SSIs from the suite [24], we opted to pilot Project Care UK in the United Kingdom first. The rationale for this was that in our formative work with adolescent advisors from 3 different groups, they consistently told us that self-kindness is a challenge for the current generation of adolescents. Furthermore, Project Care received favorable ratings on the Program Feedback Scale (PFS) in terms of its helpfulness and message, as compared to the other SSIs tested within the suite [22]. Therefore, we sought to leverage Project Care as an existing self-kindness and self-compassion SSI from the United States for use in the United Kingdom. In addition to this being a desired focus, the evidence indicates that this activity was well received by American adolescents and addressing self-hate and self-criticism may help to alleviate psychological distress as these are known risk factors for the onset and persistence of various emotional problems [27,28]. Learning to be more self-compassionate might support adolescents to achieve more positive outcomes as compared to completing cognitive and behavioral therapeutic tasks alone [29]. This learning can help people generate feelings of safety and contentment while reducing shame, self-criticism, and self-hate that might underlie and exacerbate negative cognitive processes and behaviors [30].

There has been 1 previous pilot study of a digital SSI that included both growth mindset and compassion principles and was delivered through schools in the United Kingdom [31]. The feasibility and acceptability outcomes were promising, with young people offering positive feedback about the intervention. In addition, in the treatment group, preintervention and post intervention changes in personality mindset, psychological flexibility, self-compassion, and self-esteem were observed, and small reductions in anxiety and depression were observed at the 4-week follow-up. However, the study did not assess the efficacy of the SSI; the intervention was a video, and it was only piloted in 80 UK adolescents aged between 16 and 18 years.

### Objectives

We aimed to pilot Project Care UK in a larger sample of adolescents, aged between 13 and 18 years, living in the United Kingdom. Specifically, we sought to test the feasibility, acceptability, and preliminary efficacy of the SSI to see whether this brief, scalable intervention based on evidence-based treatment is suitably promising to warrant a larger-scale randomized controlled trial in the United Kingdom. We were also keen to explore how and where to let adolescents know about Project Care UK to inform future scale-up efforts. Specifically, our research questions were as follows:

1. How do adolescents find out about Project Care UK?
2. How many adolescents complete each stage of the study, including informed consent, preintervention measures, the intervention, and postintervention measures?
3. Do adolescents view Project Care UK as an acceptable intervention?
4. Do adolescents who complete the Project Care UK intervention show improvements in short-term proximal outcomes, including hope, hopelessness, negative beliefs about self-compassion, and attitudes toward help seeking?

While research questions 1 to 3 were exploratory, for research question 4, we hypothesized preintervention to postintervention improvements across all outcome measures, including increased hope and more positive attitudes toward help seeking, as well as reductions in hopelessness and negative beliefs about self-compassion.

## Methods

### Design

We used a community-based, single-arm, pre-post intervention program evaluation within-subjects design. Thus, all participants were offered the same intervention immediately following baseline measures without a control group or randomization.

The study was reported in accordance with CONSORT-EHEALTH (Consolidated Standards of Reporting Trials of Electronic and Mobile Health Applications and Online Telehealth) guidelines [32]. Web-based survey elements were reported in line with the CHERRIES (Checklist for Reporting Results of Internet e-Surveys) guidelines [33] (Multimedia Appendix 1).

The study protocol was preregistered on the Open Science Framework (OSF) before data collection [34], with a statistical analysis plan [35] and subsequent revisions submitted before formal analysis [36].

### Recruitment

Adolescents aged between 13 and 18 years who were living in the United Kingdom and could communicate in English were eligible to take part in our study, Project Care UK. Recruitment spanned from June 2023 to July 2024.

Potential participants found out about Project Care UK via advertisements, which were created by young adults who were undergraduate students with input from our young people’s advisory group. These advertisements were shared in several ways. First, the research team posted the advertisements regularly (several times a week) on various social media channels (eg, Instagram [Meta Platforms, Inc], X [formerly known as Twitter; X Corp], and Threads [Meta Platforms, Inc]), with most activity focusing on Instagram (including a paid boost post for a few days during August 2023). Social media activity continued throughout the duration of recruitment. Second, the advertisements were also shared via email mailing lists of local community-based organizations that supported adolescents, particularly during the first 3 months of recruitment. Third, from September 2023 onward, Project Care UK was advertised to participants in Merseyside on the Young Person’s Advisory Service (YPAS; a charitable organization that provides mental health support to young people aged between 5 to 25 years) website, in their support hub, and from January 2024, through 3 schools within the Liverpool Learning Partnership. Project Care UK was signposted as an additional resource by 2 UK-wide organizations that offer free mental health support to adolescents. Kooth commenced signposting in February 2024, and Shout 85258 commenced signposting in March 2024. Project Care UK was an open survey, and no contact was made with potential participants before recruitment.

### Procedure

Project Care UK was an online, anonymous, open survey hosted on Qualtrics (Qualtrics International Inc; which specializes in the distribution of online surveys) and could be completed in any location and on any internet-connected device. Participants volunteered to participate by clicking on an anonymous link or scanning the QR code in study advertisements. First, they completed screening questions (ie, age, living in the United Kingdom, and English language ability) and ticked a box to indicate where they had found out about the study. Those who did not meet the eligibility criteria were filtered out by branch logic and displayed an ineligibility message, which signposted other sources of support. Additional measures of fraud detection were also incorporated into the study, including a built-in reCAPTCHA score and a function to prevent multiple submissions, though no cookies or IP address checkers were used. These were used as part of the data cleansing protocol to determine eligibility.

All eligible potential participants advanced to the information sheet and consent form process, with branch logic applied to route them toward the appropriate consent process based on their age (refer to the Materials section). In the information sheet, participants were informed of the purpose of the study, how long it would take, how their data would be stored, and who the principal investigator was. Participants were also reminded that their participation was voluntary and that they could withdraw at any time without giving a reason. Consenting participants then completed the preintervention measures, followed by the intervention itself. Within this, they could choose to read the information and testimonies or use the audio voice-over (this played automatically, but if participants did not want it, they could mute the sound on their device), and they completed the activities. Immediately after completing the intervention, participants completed a program feedback questionnaire and postintervention measures. Participants did not have the option to go back and review their answers. Finally, participants received debriefing information on sources of support they could access. The entire study, including the intervention, was completed within a single sitting and took approximately 30 to 40 minutes to complete (5-10 min for the preintervention measures, 15-20 min for the intervention, and 5-10 min for postintervention measures and debrief). The minimum number of questions that participants completed (eg, participants aged 16-18 y) was 136 across 68 pages, while the maximum number of questions that participants completed (eg, participants aged 13-15 y who failed to complete the Gillick competence multiple-choice questions twice and provided parental consent) was 153 questions across 74 pages. All survey questions were presented in a nonmandatory format, meaning participants were not required to respond to continue with the survey. All measures were presented in a set order (ie, questions were not randomized), and all data collected as part of the survey were stored in accordance with general data protection regulations in online drives dedicated to the project.

For those who found out about Project Care UK from YPAS and schools in the Liverpool Learning Partnership, we created a separate Qualtrics project as a copy of the main Project Care UK project. The full project used the same design, layout, and measures as Project Care UK, and the intervention itself was identical; however, participants could opt in to the research study (requiring them to complete the pre- and postintervention measures) or just access the intervention only. Therefore, this version used different branching logic to allow participants to choose which pathway they would like to complete. Additional changes were also made to debrief materials so that local sources of support (specific to the local Merseyside area) were listed for participants. In addition, the eligibility criteria to access the intervention were widened to allow individuals aged between 11 and 25 years to complete the intervention (which is more reflective of the age group that YPAS offers support and services to). Of those, for the current analysis, we only included those who opted in and provided consent to the research study and were aged between 13 and 18 years. All participants who accessed and completed the full study within the YPAS version of Project Care UK completed the same steps as earlier. Those who chose to complete the intervention only completed this and received a debrief (including local sources of support), and their data were not recorded.

### Ethical Considerations

Ethics approval was granted by the University of Bath Psychology Research Ethics Committee in May 2023 (23-061), with subsequent amendments approved by the University of Bath Research Ethics Committee. To participate in Project Care UK, informed consent was sought through a detailed information sheet and consent process (refer to the Procedure and Materials sections for further details), and participants were reminded of their right to withdraw through closing their internet browser at any point if they wished to stop taking part. All data provided were anonymous. Data safety has been ensured through adherence to General Data Protection Regulations, storing all data online, on password protected drives, and data being made accessible to members of the research team only. In addition, participants were offered the opportunity to enter a prize draw for a £50 (US $62.87) Amazon voucher. To maintain their anonymity, those who wanted to be included in the prize draw clicked a link to a separate Qualtrics project where they could leave their contact details.

### Materials

#### The Intervention: Project Care SSI

Project Care teaches adolescents about self-compassion and how being kinder to themselves could have a positive impact on their well-being, social, and academic outcomes [22,23]. Project Care was designed with input from adolescents using the BEST design elements, which are as follows: B stands for brain science or psychoeducation to teach the key concept in the program, E stands for empower the adolescent by asking them to take a “helper” or “expert” perspective, S stands for saying-is-believing exercises to consolidate learning, and T stands for testimonials and lived experience accounts from valued others [37].

Thus, Project Care SSI contains evidence-based information about how self-kindness can be helpful as well as real-world testimonies from adolescents and experts who had struggled with self-compassion and how they have achieved better outcomes since learning to be more self-compassionate. The intervention also incorporates evidence-based strategies to help overcome common barriers to self-compassion, such as recognizing that compassion extended to others can also be directed inward [30,37]. It includes activities designed to encourage reflection, support the development of a personal action plan, and consolidate learning by having participants summarize key ideas as if teaching them to someone else.

For the version we used in this study, which we refer to as “Project Care UK,” we obtained the American version of Project Care or Project Teen Goals as a Qualtrics project, which has been tested before in American adolescents [38]. With input from our young people’s advisory group and undergraduate students, we made several surface-level adaptations to the content to make it more suitable for UK adolescents (eg, changes to American language, spelling, and references such as “school grades”). Refer to Multimedia Appendix 2 for a copy of our intervention.

#### Consent Materials

For those who met the eligibility criteria, branch logic in Qualtrics was used to create consent procedure pathways for different participants based on their age. From June 2023 to January 2024, there were 2 branches: (1) all participants aged between 13 and 15 years required parental consent, and (2) participants aged between 16 and 18 years provided consent for themselves. Subsequently, to give those aged <16 years an option that did not require asking for parental consent, given that this could be a barrier to participation [39], we added an additional option whereby those aged 13 to 15 years could opt to complete additional questions to demonstrate Gillick competence and thereby consent for themselves (Multimedia Appendix 3).

### Measures

#### Overview

All measures were self-reported. In the preintervention phase, we measured demographic and clinical characteristics, including self-reported depression and anxiety symptoms, as well as hope, hopelessness, beliefs about self-compassion, and attitudes toward help seeking. Immediately, in the postintervention phase, measures of hope, hopelessness, beliefs about self-compassion, and attitudes toward help seeking were reassessed. Participants also reported views on the acceptability and feasibility of the intervention.

#### Demographics

Participants completed demographic questions related to age, sex assigned at birth, gender identity, sexual orientation, ethnicity, and subjective socioeconomic status. The item related to age allowed participants to report their age in a free text box, while all other questions asked participants to choose a response from a list that best represents their identity and circumstances. Participants completed demographic questions on sex assigned at birth, gender identity, sexual orientation, and ethnicity. Sex assigned at birth had 4 response options: “male,” “female,” “prefer not to say,” and “other”; only a single response was allowed. Gender identity included 16 options representing a range of masculine, feminine, trans, and nonbinary identities, along with “unsure” and allowed multiple selections. Sexual orientation offered 10 response options based on the UK 2021 census, along with options for those unsure or who preferred not to use a label; this question allowed 1 response. Ethnicity included six subcategories, namely White (4 options), mixed or multiple ethnic backgrounds (4 options), Asian (5 options), Black (4 options), Arab, and others, comprising 19 options in total, with 1 response permitted. Options reflected the listed categories of the 2021 census [40]. Finally, subjective social status was measured on the MacArthur Scale of Subjective Social Status–youth version (MacArthur SSS Scale–youth version) [41]. Participants responded by placing themselves on the ladder (scored 1-10) based on where they perceived their social status in the country and within their school. Scores were summed and averaged to give an overall perceived MacArthur SSS-youth scale score for each participant.

#### Measures of Mood and Internalizing Symptoms

The Patient Health Questionnaire–2 items (PHQ-2) [42] is a brief self-report screening tool for depressive symptoms over the previous 2 weeks. Items (eg, “little interest or pleasure in doing things”) are rated on a 4-point Likert scale ranging from 0 (“not at all”) to 3 (“nearly every day”). A higher score on the PHQ-2 indicates greater depressive symptoms. Furthermore, this measure appears to have acceptable internal reliability and validity in adolescents [42-45].

The Generalized Anxiety Disorder–2 items (GAD-2) checklist [46] is a brief self-report screening tool for generalized anxiety over the previous 2 weeks. Items (eg, “feeling nervous, anxious, or on edge”) are scored on a 4-point Likert scale ranging from 0 (“not at all”) to 3 (“nearly every day”). A higher score on GAD-2 indicates greater generalized anxiety symptoms. In addition, this measure has been found to have good internal reliability and validity [46].

The State Hope Scale (SHS) [47] is a 6-item self-report scale that measures hope and optimism around one’s goals. Items are split into 2 subscales, *agency* items (eg, “at present, I am energetically pursuing my goals”) refer to one’s ability to start and complete actions, and *pathway* items (eg, “I can think of many ways to reach my current goals”) refer to the routes available to reach one’s goals. Items are rated on an 8-point Likert scale ranging from 0 (“definitely false”) to 8 (“definitely true”). Higher scores indicate greater hope. In addition, the SHS appears to have good internal reliability and concurrent and discriminant validity [47]. It must be noted that participants were presented with all 6 items before intervention, while after intervention, they were presented with items 1, 3, and 5, which make up the “pathways” subscale.

The Beck Hopelessness Scale (BHS) [48] is a 4-item self-report scale that measures hopelessness as a cognitive trait of depression. Items pose statements about the self, world, and future (eg, “I look forward to the future with hope and enthusiasm”) and ask participants to rate their agreement with each statement on a 4-point Likert scale ranging from 0 (“absolutely disagree”) to 3 (“absolutely agree”). This measure has been shown to have excellent internal reliability and good validity [48].

The Beliefs About Self-Compassion Scale (BSCS) [49] is a 10-item self-report measure that assesses an individual’s beliefs about self-compassion and how it might have a negative impact on them if they were to show self-compassion. Items (eg, “When I am kind to myself, I will behave more self-indulgently”) are rated on a 5-point Likert scale ranging from 1 (“strongly disagree”) to 5 (“strongly agree”). A higher score suggests greater negative beliefs about self-compassion.

An adapted version of the General Help-Seeking Questionnaire (GHSQ) [50] was used. This contains 1 item, which asks participants to rate how likely they are to seek mental health support from the 9 listed sources. This was rated on a 7-point Likert scale from 1 (“extremely unlikely”) to 7 (“extremely likely”). To make the questionnaire culturally and developmentally relevant for UK youth, “online (eg, social media, forums)” was added as an optional source, while “intimate partner” and “minister or religious leader” were omitted. Participants could also identify other sources of support that are not listed. The original measure has been shown to have satisfactory internal reliability and validity [50].

#### Measures of Intervention Satisfaction

The PFS [51] used in this study has been adapted from existing validated feedback scales and is routinely used to evaluate the acceptability and feasibility of SSIs. Items (eg, “would recommend to a friend”) are rated on a 5-point Likert scale ranging from 1 (“really disagree”) to 5 (“really agree”). Participants could also offer additional feedback and opinions about the activity in 3 open-ended questions.

### Statistical Analysis

Data were cleaned according to the study’s eligibility criteria and analyzed using the R programming language (version 4.3.1; R Foundation for Statistical Computing) [52] within the RStudio integrated development environment (version 2024.09.1) [53]. The statistical analysis plan was preregistered on the OSF [35], with minor revisions made during the analysis phase [36].

*Feasibility* was examined by calculating counts (n) and proportions (percentages) of participants completing each stage of the study, including informed consent, preintervention measures, the intervention, and postintervention measures. Completeness checks, which defined participant flow, were conducted following the submission of each questionnaire. No statistics on view rate were collected.

Reach and adoption were assessed by summarizing demographic variables (age, sex assigned at birth, sexual orientation, ethnicity, and perceived social status) and clinical characteristics (GAD-2 and PHQ-2). Continuous variables were reported using means and SDs, while categorical variables were summarized using counts and percentages.

*Implementation*, in terms of recruitment sources, was evaluated by calculating the number and percentage of participants recruited through each channel (eg, well-being organizations, social media platforms, community email lists, and schools).

*Acceptability* was assessed using central tendency (means) and dispersion (SDs) for the full-scale and item-level scores on the PFS. A score of ≥3.0 (or ≥21.0 total) was preregistered as the threshold for “at least acceptable,” corresponding to a neutral average response. A higher threshold of ≥3.5 (or ≥24.5 total), commonly applied in previous literature [51], was used to indicate a “favorable” rating. The proportion of participants meeting each threshold was reported at both item and scale levels.

*Preliminary efficacy* examined the preintervention to postintervention changes in well-being outcomes, including the SHS pathways subscale, BHS, BSCS, and GHSQ. While the original preregistered analysis plan [48] specified the use of 1-tailed paired *t* tests, an unexpectedly large recruited sample, combined with substantial attrition and variability in data completeness, warranted the use of linear mixed-effects models as a more appropriate and robust analytic approach. The revised analysis plan is available on OSF [36], and results from the original paired *t* test analyses are reported in Multimedia Appendix 4 for transparency.

For the revised analysis plan, linear mixed-effects models were fitted using the *lme4* package in R [54]. Models included a fixed effect of time (before intervention vs after intervention) and a random intercept for participant ID to account for within-subject variations. Outcomes included the SHS pathways subscale, BHS, BSCS, and GHSQ.

Using the *MissMech* package in R [55], a nonparametric test for missing completely at random indicated that the missingness mechanism was not related to either observed or unobserved variables (*P*=.28). This supported the use of complete case analysis for the primary models, which included only participants with both pre- and postintervention data. To assess robustness, a sensitivity analysis was conducted using an intention-to-treat (ITT) approach, where missing postintervention data were imputed using the *mice* package in R [56].

For all models, β coefficients, *P* values (significance set at *P*<.05), and standardized effect sizes (Cohen *d*) were reported. Effect sizes were interpreted according to standard benchmarks: small (0.2), medium (0.5), and large (0.8) [57]. The proportion of participants showing improvement from before intervention to after intervention was reported, with improvement defined as increased scores on the SHS and GHSQ and decreased scores on the BHS and BSCS.

It should also be noted that, although the original analysis plan included all 6 items from the SHS, only the pathways subscale (items 1, 3, and 5) was administered. This reflected a focus on proximal cognitive outcomes likely to change within an immediate pre-post design, whereas agency items (2, 4, and 6), which assess behavioral goal pursuit, may require longer-term follow-up to detect change. In the GHSQ, the “Shout85285” help-seeking source was incorrectly encoded and therefore excluded from pre-post comparisons. In addition, the gender identity variable was omitted from analyses due to a survey coding error that rendered the data unusable.

## Results

### Feasibility

**Figure 1:**
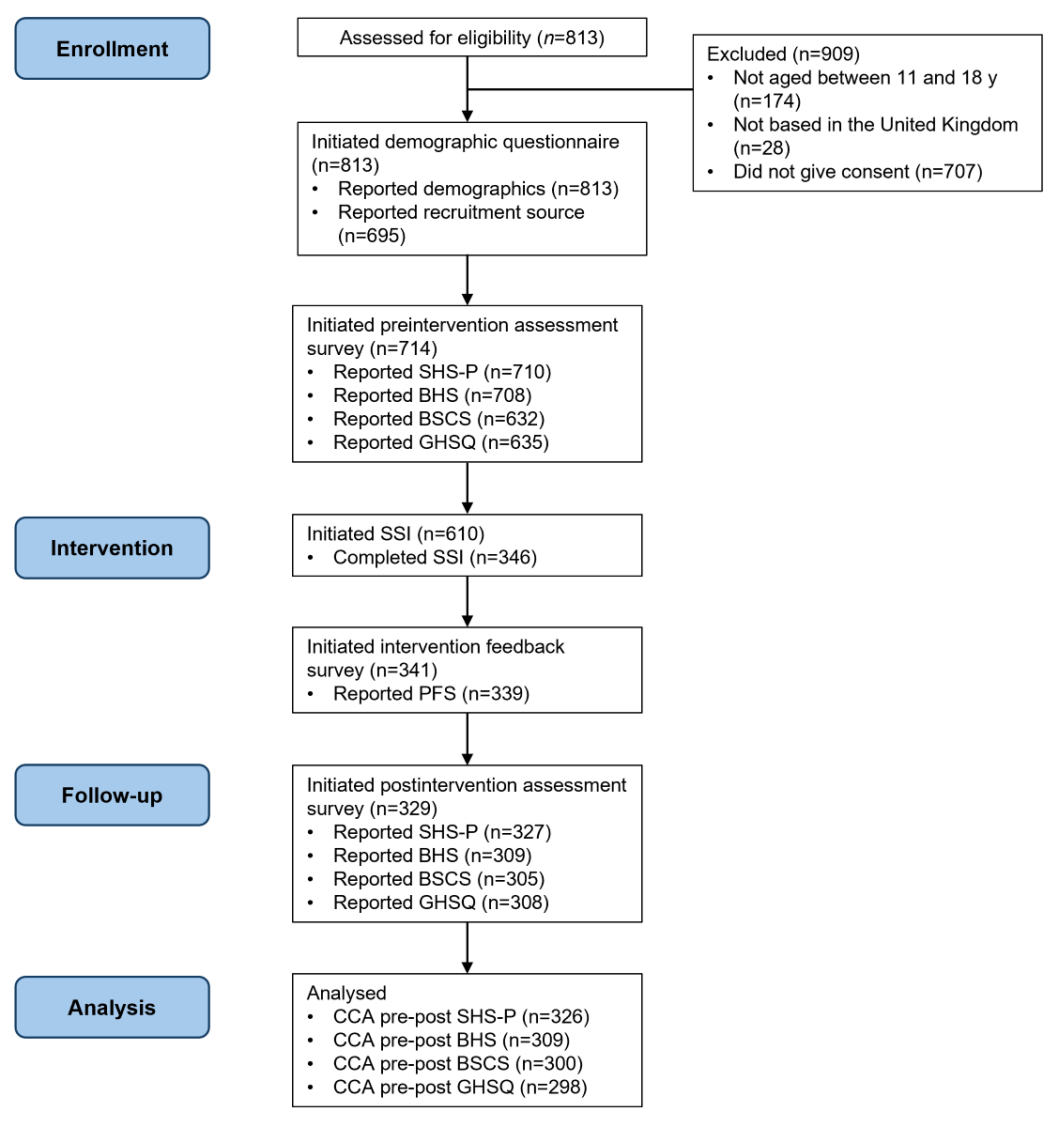
Participant flow diagram. This modified CONSORT-EHEALTH (Consolidated Standards of Reporting Trials of Electronic and Mobile Health Applications and Online Telehealth) flow diagram depicts the number of participants at each study phase, including enrollment, intervention allocation, follow-up, and analysis. BHS: Beck Hopelessness Scale; BSCS: Beliefs About Self-Compassion Scale; CCA: complete case analysis; GHSQ: General Help-Seeking Questionnaire; PFS: Program Feedback Scale; SHS-P: State Hope Scale pathways; SSI: single-session intervention.

A total of 1722 individuals were assessed for eligibility, of whom 813 (47.21%) met the criteria and provided consent to participate. Among these, all 813 (100%) participants initiated the demographic questionnaire, 714 (87.8%) initiated the preintervention assessment survey, 610 (75%) initiated the SSI, 341 (41.9%) initiated the intervention feedback survey, 329 (40.5%) initiated the postintervention assessment survey, and 304 (37.4%) completed the study. For the preliminary efficacy analysis, complete case data were available for 326 (40.1%) participants for the SHS pathways subscale, 309 (38%) participants for the BHS, 300 (36.9%) participants for the BSCS, and 298 (36.7%) participants for the GHSQ. Participant flow is detailed in Figure 1.

Dropout was relatively high in terms of both *overall study attrition* (509/813, 62.6% of the participants left the study early) and *intervention attrition* (264/610, 43.3% of the participants left the SSI early).

There was substantial variability in SSI completion times, from 9 minutes 17 seconds to 173 minutes 39 seconds (mean 23 min 39 s, SD 15 min 11 s).

### Reach and Adoption

**Figure 2:**
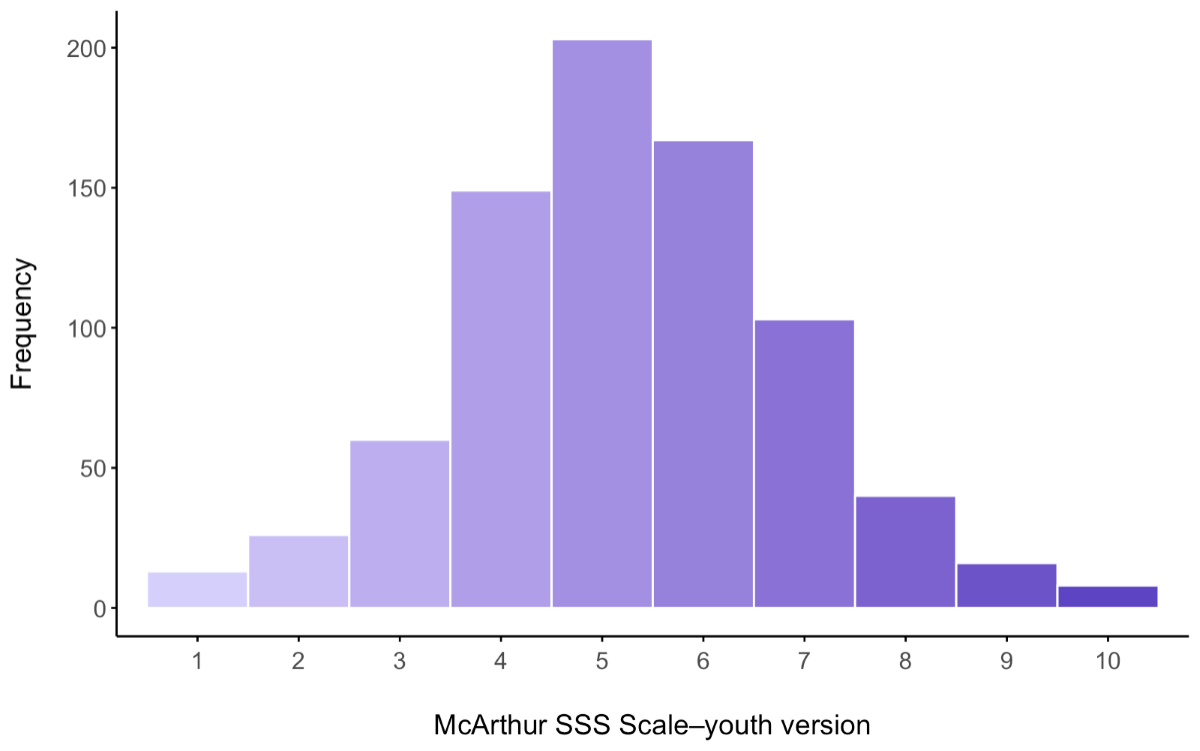
Histogram depicting the distribution of subjective social status (SSS). This histogram illustrates the distribution of subjective social status scores as measured by the MacArthur Scale of Subjective Social Status–youth version. Each bar represents the frequency of respondents with a specific score ranging from 1 to 10. Gradient color indicates the progression of scores from light purple (“low subjective social status”) to dark purple (“high subjective social status”).

A total of 813 participants initiated the demographic questionnaire. They were aged between 13 and 18 years (mean 15.38, SD 1.58 y), with most being assigned female sex at birth (n=699, 86%), White (n=611, 75.2%), and not identifying as heterosexual (466/809, 57.6%). Participants indicated that their family’s subjective social status was average relative to other UK households (mean 5.25, SD 1.67; Figure 2).

Mental health difficulties were evident among the participants, with 71.5% (537/751) of the participants meeting or exceeding the threshold for elevated depression symptoms (ie, PHQ-2 scores ≥3) and 75.8% (560/739) of the participants for elevated anxiety symptoms (GAD-2 scores ≥3).

Demographic distributions were broadly similar between those who initiated the demographic form and those who completed the full study. However, while missing completely at random analyses did not indicate systematic sources of selective attrition, there was a noticeable descriptive drop in the proportion of participants identifying as heterosexual (from 343/809, 42.4% to 110/302, 36.4%). Comprehensive demographic information is provided in Table 1.

**Table 1:** Demographic information.

Demographic characteristics		Initiated	Completed
Age (y), mean (SD)		15.38 (1.58)^a^	15.36 (1.53)^b^
**Sex assigned at birth, n/n (%)**			
	Female	699/813 (86)	259/304 (85.2)
	Male	100/813 (12.3)	39/304 (12.8)
	Other birth sex	2/813 (0.2)	2/304 (0.7)
	Prefer not to say	12/813 (1.5)	4/304 (1.3)
**Sexual orientation, n/n (%)**			
	Heterosexual	343/809 (42.4)	110/302 (36.4)
	Homosexual	86/809 (10.6)	34/302 (11.3)
	Bisexual	150/809 (18.5)	57/302 (18.9)
	Pansexual	30/809 (3.7)	11/302 (3.6)
	Queer	15/809 (1.9)	9/302 (3)
	Asexual	27/809 (3.3)	16/302 (5.3)
	Unsure or questioning	77/809 (9.5)	34/302 (11.3)
	Does not use a label	46/809 (5.7)	14/302 (4.6)
	Other sexual orientation	19/809 (2.3)	12/302 (4)
	Prefer not to say	16/809 (2)	5/302 (1.7)
**Ethnicity, n/n (%)**			
	Asian or Asian British	75/803 (9.2)	30/303 (9.9)
	Black or Black British	48/803 (6)	17/303 (5.6)
	White	611/803 (76.1)	227/303 (74.9)
	Mixed ethnic group	52/803 (6.5)	22/303 (7.3)
	Other ethnic group	17/803 (2.1)	7/303 (2.3)
Social status score, mean (SD)		5.25 (1.67)^c^	5.24 (1.53)^d^
**Depression**			
	PHQ-2^e^ score, mean (SD)	3.81 (1.75)^f^	3.72 (1.75)^d^
	Clinical cases, n/N (%)	537/751 (71.5)	213/303 (70.3)
**Anxiety**			
	GAD-2^g^ score, mean (SD)	4.08 (1.83)^h^	4.03 (1.78)^b^
	Clinical cases, n/N (%)	560/739 (75.8)	232/304 (76.3)

^a^n=818.

^b^n=304.

^c^n=785.

^d^n=303.

^e^PHQ-2: Patient Health Questionnaire–2 items.

^f^n=751.

^g^GAD-2: Generalized Anxiety Disorder–2 items.

^h^n=739.

### Implementation: Recruitment Sources

A total of 695 (85.5%) of the 813 participants reported where they had heard about the Project Care UK study. For these 695 participants, third-party well-being organizations were the most common recruitment sources, accounting for 662 (95.3%) participants, with Kooth being the largest individual contributor (n=603, 86.8%). In contrast, recruitment via social media platforms was minimal (n=14, 2%), while community email distributions (n=1, 0.1%) and secondary education institutions (n=0, 0%) contributed few to no participants. In addition, 18 (2.6%) participants reported discovering the study through alternative sources. Recruitment source distributions were similar between those who initiated the survey and those who progressed to the end of the study. A detailed summary of the study’s recruitment sources is provided in Table 2.

**Table 2:** Recruitment sources of the Project Care UK study.

Recruitment source		Initiated (n=695), n (%)	Completed (n=258), n (%)
**Well-being organizations**			
	Kooth	603 (86.8)	230 (89.1)
	Shout 85285	44 (6.3)	14 (5.4)
	YPAS^a^	15 (2.2)	8 (3.1)
**Social media platforms**			
	Instagram	9 (1.3)	2 (0.8)
	Threads	1 (0.1)	0 (0)
	Twitter (subsequently rebranded X)	1 (0.1)	0 (0)
	TikTok	2 (0.3)	0 (0)
	YouTube	1 (0.1)	0 (0)
Community email distributions		1 (0.1)	0 (0)
Secondary education institutions		0 (0)	0 (0)
Other sources		18 (2.6)	4 (1.6)

^a^YPAS: Young Person’s Advisory Service.

### Acceptability

**Figure 3:**
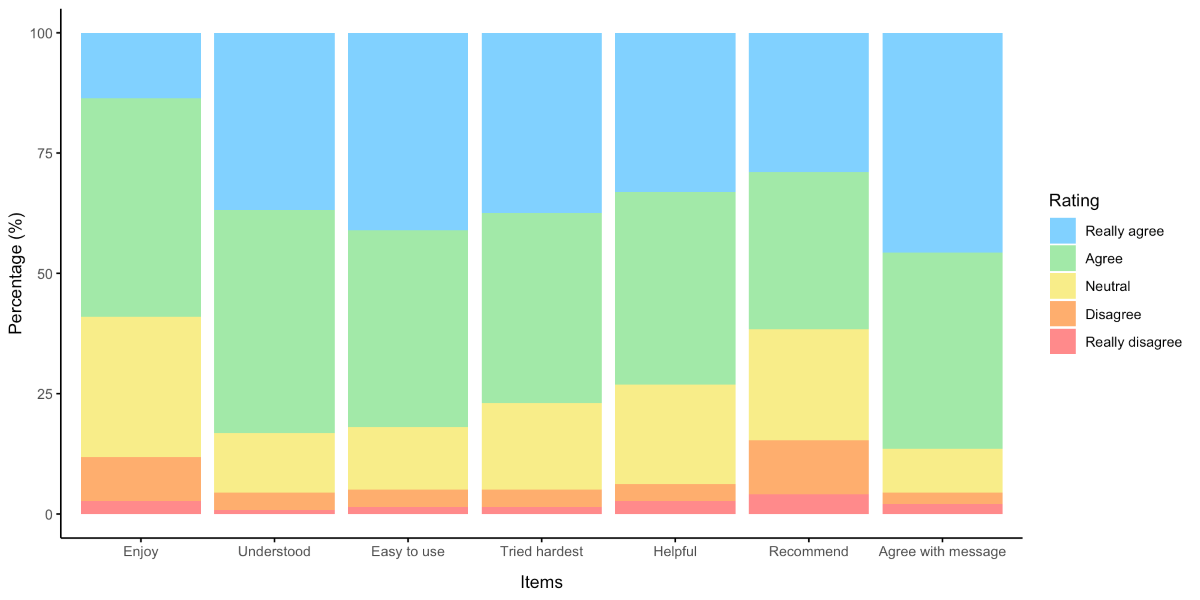
Stacked bar chart depicting the distribution of ratings across each item of the Program Feedback Scale. Together, blue (ie, “really agree”), green (ie, “agree”), and yellow (ie, “neutral”) denote an endorsement of the given item as “at least acceptable.”.

Of the 813 participants, 341 (41.9%) initiated the intervention feedback survey and 339 (41.7%) completed it. Summed scale scores of these 339 completers indicated that the SSI was well-received, with 313 (92.3%) participants endorsing it as “at least acceptable” (ie, PFS full-scale scores ≥21.0) and 271 (79.9%) endorsing it as “favorable” (ie, PFS full-scale scores ≥24.5). For individual PFS domains, 299-324 (88.2%-95.6%) of participants endorsed each domain as “at least acceptable” (ie, PFS domain scores ≥3.0) and 200-282 (59%-83.2%) endorsed each domain as “favorable” (ie, PFS domain scores ≥3.5; Table 3 and Figure 3).

**Table 3:** Ratings for each item on the Program Feedback Scale (PFS; N=339)^a^.

Item content	Ratings, mean (SD)	“At least acceptable” endorsement (≥3.0), n (%)	“Favorable” endorsement (≥3.5), n (%)
Enjoy	3.58 (0.93)	299 (88.2)	200 (59)
Understood	4.15 (0.83)	324 (95.6)	282 (83.2)
Easy to use	4.17 (0.89)	322 (95)	278 (82)
Tried hardest	4.08 (0.91)	322 (95)	261 (77)
Helpful	3.97 (0.96)	318 (93.8)	248 (73.2)
Recommend to a friend	3.71 (1.12)	287 (84.7)	209 (61.7)
Agree with the message	4.26 (0.87)	324 (95.6)	293 (86.4)
Full scale	27.91 (4.74)	313 (92.3)	271 (79.9)

^a^This table presents the mean scores and their corresponding SDs for the full scale and each individual item of the (PFS). In addition, it reports the counts (n) and percentages of individuals who endorsed Project Care UK as “at least acceptable” and “favorable.” The intervention is endorsed as “at least acceptable” overall if the full-scale score is ≥21.0 and per domain if individual item scores are ≥3.0. The respective cutoffs for a “favorable” endorsement are ≥24.5 for the full scale and ≥3.5 for each item.

### Preliminary Efficacy: Main Analyses

Of the 813 participants, 329 (40.5%) initiated the postintervention assessment survey. Statistically significant improvements were observed for 3 of the outcome measures, including increased hope (326/813, 40.1%; *P*<.001; Cohen *d*=0.72), decreased hopelessness (309/813, 38%; *P*<.001; Cohen *d*=–0.73), and decreased negative beliefs about self-compassion (300/813, 36.9%; *P*<.001; Cohen *d*=–0.64).

**Figure 4:**
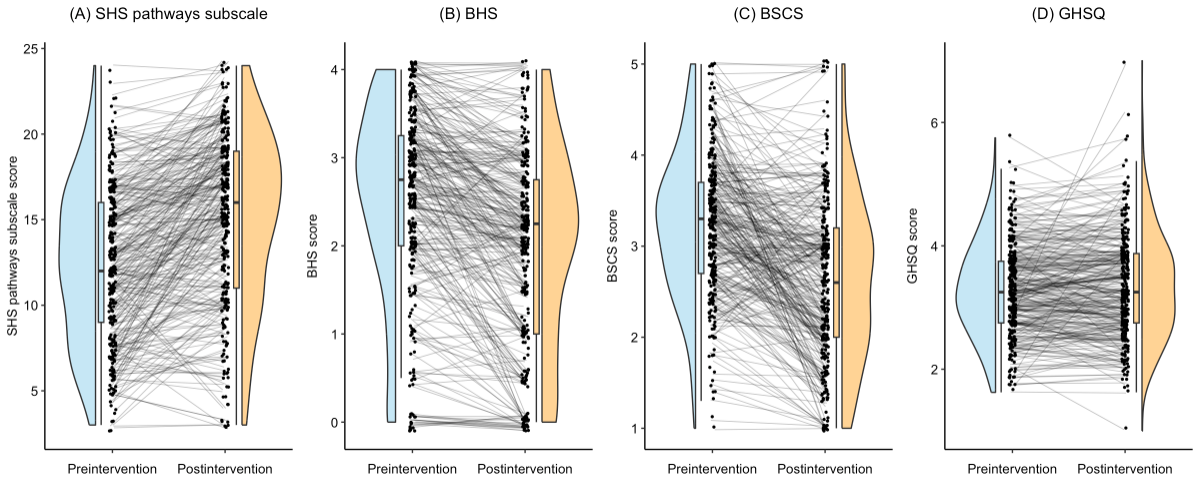
Modified rain cloud plots depicting pre-post intervention comparisons of hope, hopelessness, self-compassion, and help seeking. The integrated plots comprehensively display pre-post data for hope, hopelessness, self-compassion, and help seeking. Graphs include pre-post trajectories for each participant (ie, individual data points and connecting lines), central tendencies (box plots), and frequency distributions (rain cloud plots). BHS: Beck Hopelessness Scale; BSCS: Beliefs About Self-Compassion Scale; GHSQ: General Help-Seeking Questionnaire; SHS: State Hope Scale.

For those cases with complete data, rates of individual-level improvement were also significant, with 69.9% (228/326) of the participants showing an improvement in hope, 88% (272/309) of the participants showing an improvement in hopelessness, and 77% (231/300) of them showing an improvement in negative beliefs about self-compassion. Help-seeking tendencies did not exhibit any significant change (298/813, 36.7%; *P*=.13; Cohen *d*=0.09). Detailed pre- and postintervention comparisons are presented in Table 4 and Figure 4.

**Table 4:** Pre- and postintervention comparisons of hope, hopelessness, self-compassion, and help-seeking^a^.

Scale	Preintervention assessment, mean (SD)	Postintervention assessment, mean (SD)	β (SE)	*t* test (*df*)	*P* value	Cohen *d* (95% CI)	Improved cases, n (%)
SHS^b^ pathways subscale	12.30 (4.55)	15.02 (5.02)	2.72 (0.21)	13.07 (325)	<.001	0.72^c^ (0.60 to 0.85)	228 (69.9)^d^
BHS^e^	2.54 (1.07)	1.99 (1.13)	–0.56 (0.04)	–12.92 (308)	<.001	–0.73^c^ (–0.86 to –0.61)	272 (88)^f^
BSCS^g^	3.23 (0.78)	2.65 (0.90)	–0.58 (0.05)	–11.12 (299)	<.001	–0.64^c^ (–0.77 to –0.52)	231 (77)^h^
GHSQ^i^	3.30 (0.75)	3.35 (0.86)	0.05 (0.03)	1.54 (297)	<.13*	0.09^j^ (–0.02 to 0.02)	133 (44.6)^k^

^a^Preintervention and postintervention comparisons for hope (State Hope Scale pathways subscale), hopelessness (Beck Hopelessness Scale), beliefs about self-compassion (Beliefs About Self-Compassion Scale), and help-seeking (General Help-Seeking Questionnaire). Values represent the number of cases, percentages, means, SDs, fixed effect estimates (β), *dfs*, *t* test values, *P* values, effect sizes (Cohen *d*) with 95% CI, and percentages of participants who improved (improved cases). Participants showing improvement in outcome measures (improved cases) were also reported, defined as increased scores on the State Hope Scale and General Help-Seeking Questionnaire and decreased scores on the Beck Hopelessness Scale and Beliefs About Self-Compassion Scale. Effect sizes are classified as small, medium, or large. Pre-post changes in hope, hopelessness, and beliefs about self-compassion were significant.

^b^SHS: State Hope Scale.

^c^Medium effect size.

^d^n=326.

^e^BHS: Beck Hopelessness Scale.

^f^n=309.

^g^BSCS: Beliefs About Self-Compassion Scale.

^h^n=300.

^i^GHSQ: General Help-Seeking Questionnaire.

^j^Negligible effect size.

^k^n=298.

### Preliminary Efficacy: Sensitivity Analyses

To assess the robustness of the main findings for preliminary efficacy, a sensitivity analysis using an ITT approach was conducted. In this analysis, missing postintervention values were imputed using multiple imputation procedures as outlined in the revised statistical analysis plan [36]. Results from the ITT analyses were consistent with the complete case analyses: statistically significant improvements were observed in hope (709/813, 87.2%; *P*<.001), hopelessness (708/813, 87.1%; *P*<.001), and negative beliefs about self-compassion (632/813, 77.7%; *P*<.001), with no statistically significant change in help-seeking intentions (635/813, 78.1%; *P*=.19). Full inferential results are presented in Multimedia Appendix 5.

## Discussion

### Principal Findings

Our single-arm pre-post program evaluation of a minimally adapted version of the American SSI Project Care in the United Kingdom showed that recruitment through large-scale third-party well-being organizations was the most effective implementation strategy. In total, 37.4% (304/813) of our participants completed the study, and most participants who discontinued did so during the SSI. Most of our participants were assigned female sex (699/813, 86%) at birth, were White (611/813, 75.2%), and did not identify as heterosexual (466/809, 57.6%). Overall, participants viewed Project Care UK as acceptable and offered favorable ratings for the SSI. In addition, participants reported significant improvements in hope, hopelessness, and self-compassion. No significant differences were found in help seeking.

A key uncertainty that we need to address to capitalize on digital self-help SSIs, such as Project Care, at the public health level in the UK context is how to let adolescents know about the availability of these interventions. In our study, we piloted several different recruitment routes. As previous research studies and open-access trials in the United States by the Lab for Scalable Mental Health have predominantly reached adolescents via posts and targeted advertisements on social media, particularly Instagram [24], we anticipated that most adolescents would be recruited via social media [58]. This is also consistent with evidence that adolescents look for health information generally [58,59] and mental health information specifically online [60]. Furthermore, in our formative work in the United Kingdom, adolescents identified that they tend to look for support on the internet and social media when they first begin to struggle with their mental health [61]. However, contrary to our expectations, only a very small number of our participants (14/695, 2%) were recruited via social media. There are several potential explanations for this. It may be because our social media accounts do not have the adolescent following that those from the Lab for Scalable Mental Health do, and therefore our advertisements did not reach the intended target audience or that there has been a shift away from social media since the US-based studies. It could also be that in the United Kingdom, where health care, including mental health care, is free to access, the offer of free mental health help is less persuasive and attractive than in the United States, where health care is generally charged to the user. While adolescents spend time on social media, they may doubt the trustworthiness of information they find there [62] and might therefore be cautious about taking part in studies advertised there.

During the recruitment phase of our study, we introduced several alternative recruitment routes and found that most of our participants (603/695, 86.8%) heard about Project Care UK through a large-scale third-party mental health organization. Kooth is commissioned in most of England to provide free, anonymous online mental health help on demand, such as activities to manage emotions, psychoeducation, and crisis support, which is largely used by adolescents who may be struggling with mental health difficulties [63]. Adolescents in the United Kingdom have previously identified that they would prefer to use reputable and trusted sources of mental health support [50,51]. Therefore, they may have been more likely to engage with Project Care when it was listed as a resource in organizations that they already knew of and trusted, and this trust appears to have been extended to Project Care when it was signposted there. There are multiple similarities between self-help SSIs such as Project Care and organizations such as Kooth and Shout, which offer on demand, convenient, and anonymous information and support, and users are not necessarily required to seek adults’ permission (eg, parental consent and general practitioner referral), all of which are known barriers to accessing mental health support [7,10]. Hence, those seeking help from these organizations might also be particularly likely to view SSIs favorably due to these shared characteristics in terms of how help is provided. Thus, embedding SSIs within the support offered by these organizations could be a way to extend on what they currently provide.

### Limitations and Future Directions

Many (509/813, 62.6%) participants did not complete the study. It may be that while the Project Care UK SSI did not fit with what some adolescents were looking for, offering a choice of SSIs to enable adolescents to select the one that they feel best fits their needs could keep them better engaged. Notably, in open platform trials where Project Care was offered as one of 3 SSI options, only some adolescents selected it (19% of the individuals aged 13-18 years in Project YES [23] and 29% of the individuals aged 11-17 years in Project YES San Antonio) [23]. It is also possible that the type of support that adolescents are looking for is bite-sized information (eg, TikTok-style short videos) that is easy to consume rather than longer, activity-based support (such as Project Care UK). A recent narrative review [19] reported that some of the program features that facilitate engagement in digital interventions are efficient (eg, bite sized) and interesting content (eg, interactive content or videos). A briefer version of an SSI embedded in Tumblr has been found to be feasible and acceptable in the United States [64] Alternatively, it may be that some participants discontinued because they had received what they wanted from the SSI, a pattern which has been found in a subgroup of adolescents with depression who discontinue in-person, multisession psychological therapy [65], and which does not necessarily predict unfavorable outcomes [66]. Exploring barriers to and facilitators of SSI completion using process evaluation [67] could shed light on these possibilities to guide further developments of SSIs to better meet adolescents’ needs and preferences.

Similar to samples from SSI studies in the United States [22-24], our sample was diverse, particularly regarding sexual orientation and ethnicity. For example, 9.2% (75/803) of the participants identified their ethnic group as Asian or Asian British, closely matching UK population estimates of 9.3% [68]. In addition, 57.6% (466/809) of the participants did not identify as heterosexual, which exceeds national estimates of approximately 10% [41,68], reflecting strong engagement with sexual minority adolescents. While previous research has often struggled to reach these populations [6,69], our SSI, through multiple recruitment strategies, successfully engaged both ethnic minority groups and young people who identified as nonheterosexual. Thus, SSIs, offered anonymously and on demand online, could be a promising method of reaching underserved groups and providing them with reputable, evidence-based mental health support, as an expansion on traditional clinic-based provision.

Despite this promising finding, most of our sample (ie, 699/813, 86%) were assigned female sex at birth, which is in line with figures from SSI acceptability and feasibility studies conducted in American adolescents [21]. Previous research has suggested that stigma and societal expectations of masculinity are potential barriers to male adolescents engaging with therapeutic support [70], and interventions such as Project Care UK could transcend these barriers by providing online, anonymous support. However, it remains largely unclear whether online SSIs that are offered anonymously are acceptable and feasible for male individuals, and future research that considers how to leverage this type of support among them would be beneficial.

Ours is the first evaluation of the Project Care SSI, which included a self-compassion measure. Promisingly, 77% (231/300) of our sample reported improved beliefs about self-compassion after intervention as an immediately measurable change, and future studies could look at whether this translates over time into changes in self-compassionate thinking and responding.

Even though our evidence of effectiveness on proximal outcomes and preliminary efficacy is promising, it is important to acknowledge the possibility that for some adolescents, SSIs may not be helpful, indicated by several responses (26/339, 7.7%) to the PFS in our study. Future studies could more explicitly explore adverse events and harms to understand more about those who indicate that it is unhelpful. Studies could include adverse events and harm measures such as the Negative Effects Questionnaire [71].

The overall study attrition rate (62.6%) and the SSI attrition rate (43.3%) were quite high, which could potentially limit the validity of our findings. While our data suggested that Project Care was feasible overall and across different groups of young people (eg, across ethnic groups, genders, and sexual orientations), it is unclear why some participants discontinued, and it will be important for future studies to explore this. Our conclusions apply to those participants who did complete measures but cannot be assumed to apply to those who did not. As noted in other SSI evaluations [72], about half (346/813, 42.6%) of the eligible participants who accessed Project Care UK completed the SSI.

In addition to the proximal outcome measures we used, future program evaluations of SSIs could also measure general state affect using the Positive and Negative Affect Schedule [73] as an additional proximal outcome measure. In our study, as we did not conduct a follow-up, we were unable to draw any conclusions about short- and long-term changes in mental health symptoms or self-compassion, and future studies would benefit from additional follow-up points to address this.

As this was a pilot study without a control group, it is unclear whether the observed effects were due to the effects of the intervention or the specific content of this SSI. Future work could compare the outcomes for participants randomly allocated to the Project Care SSI to those allocated to an online activity control condition to account for the time spent doing activities online, for instance, to enable more robust conclusions about the effectiveness of the specific Project Care SSI. Although given our findings of significant pre-post increases in hope and reduced hopelessness and negative beliefs about self-compassion, the Project Care intervention appears to have been effective. While it is possible that young people were able to use the intervention to learn about the value of self-compassion and how they could implement this into their lives, as demonstrated through significant pre-post reductions in negative beliefs about self-compassion, it is ultimately unclear whether the intervention achieved these pre-post changes through its intended mechanisms. Nevertheless, our findings suggest that it would be appropriate to disseminate the Project Care UK intervention, particularly as it would make free, online, evidence-based support about self-compassion widely available to UK adolescents, which they have highlighted as a priority.

### Conclusions

SSIs such as Project Care could offer a scalable solution for expanding access to early help and overcoming some of the existing barriers that adolescents face. Through leveraging large-scale third-party mental health organizations whose primary users are adolescents [63], it appears to be feasible to make SSIs such as Project Care widely available, although activities must align with the organization’s primary offers of support. Adolescents from racial and sexual minority backgrounds who are underserved by traditional clinic-based services seem to be better represented in studies of SSIs, including our own and previous studies in the United States [21], although more work is needed to establish how to engage individuals identifying as male in using these kinds of scalable supports. Our findings provide initial indications of the preliminary efficacy of Project Care in the UK context. Additional work is needed to more robustly test it by comparing it to other interventions and following up with participants over time to explore whether the effects are maintained and translated into mental health benefits beyond the proximal outcomes reported in this study.

## Appendix

Multimedia Appendix 1 CHERRIES (Checklist for Reporting Results of Internet e-Surveys).

Multimedia Appendix 2 Project Care UK intervention.

Multimedia Appendix 3 Project Care UK consent process.

Multimedia Appendix 4 Preliminary efficacy—results of preregistered data analysis.

Multimedia Appendix 5 Preliminary efficacy—sensitivity analysis.

## Abbreviations

BHS: Beck Hopelessness Scale
BSCS: Beliefs About Self-Compassion Scale
CHERRIES: Checklist for Reporting Results of Internet e-Surveys
CONSORT-EHEALTH: Consolidated Standards of Reporting Trials of Electronic and Mobile Health Applications and Online Telehealth
GAD-2: Generalized Anxiety Disorder–2 items
GHSQ: General Help-Seeking Questionnaire
ITT: intention-to-treat
OSF: Open Science Framework
PFS: Program Feedback Scale
PHQ-2: Patient Health Questionnaire–2 items
SHS: State Hope Scale
SSI: single-session intervention
YPAS: Young Person’s Advisory Service
