# Supplementary material for: Investigating Project Care UK, a Web-Based Self-Help Single-Session Intervention for Youth Mental Health: Program Evaluation
Source: JMIR Ment Health. 2025 Jun 18;12:e72077. doi: 10.2196/72077 (PMC12223457; doi:10.2196/72077)
Supplement: Multimedia Appendix 1 [file mental_v12i1e72077_app1.docx]

## **Multimedia Appendix 1: CHERRIES checklist for reporting online surveys**

Checklist for Reporting Results of Internet E-Surveys (CHERRIES)

| *Item Category* | *Checklist Item* | *Reporting* |
| --- | --- | --- |
| Design | Describe survey design | Methods Design *- We used a community-based, single arm, pre-post intervention programme evaluation within subjects’ design. Thus, all participants were offered the same intervention immediately following baseline measures, without a control group or randomisation.* |
| IRB (Institutional  Review Board) approval and informed consent process | IRB approval | Methods Ethical Permission *- Ethical permission was granted by the University of Bath Psychology Research Ethics Committee in May 2023 (reference 23-061), with subsequent amendments approved by the University of Bath’s Research Ethics Committee.* |
|  | Informed consent | Methods Procedure - *In the information sheet, participants were informed of the purpose of the study, how long it would take, how their data would be stored and who the principal investigator was. Participants were also reminded that their participation was voluntary and that they could withdraw at any time without giving reason.* |
|  | Data protection | Methods Procedure - *All data collected as part of the survey was stored in accordance with general data protection regulations in online drives dedicated to the project.* |
| Development and  pretesting | Development and testing | Methods Materials *- For the version we used in the current study, which we refer to as ‘Project Care UK’, we obtained the American version of Project Care/Project Teen Goals as a Qualtrics project, which has been tested before in American adolescents [36]. With input from our Young People’s Advisory Group and undergraduate students, we made several surface level adaptations to the content to make it more suitable for UK adolescents (e.g., changes to Americanised language, spelling, and cultural references like “school grades”).* |
| Recruitment process and description of the  sample having access to the questionnaire | Open survey versus closed survey | Methods Procedure *- Project Care UK was fully online, anonymous, open survey hosted on Qualtrics (which specialises in distribution of online surveys),and could be completed in any location and on any internet-connected device.* *Participants volunteered to participate by clicking on an anonymous link or scanned the QR code in study adverts.* |
|  | Contact mode | Methods Procedure *- Participants volunteered to participate by clicking on an anonymous link or scanned the QR code in study adverts.* |
|  | Advertising the survey | Methods Procedure *- Potential participants found out about Project Care UK via adverts which were created by young adults who were undergraduate students with input from our Young People’s Advisory Group (see online Supplementary materials S1 for an example). These adverts were shared in several ways. Firstly, the research team posted the adverts regularly (several times a week) on various social media channels (e.g., Instagram, X (formerly Twitter), Threads), with most activity focusing on Instagram (including a paid boost post for a few days during August 2023). Social media activity continued for the duration of recruitment. Secondly, the adverts were also shared via email mailing lists of local community-based organisations who support adolescents, particularly during the first 3 months of recruitment. Thirdly, from September 2023 onwards, Project Care UK was advertised to participants in Merseyside on the Young Person’s Advisory Service (YPAS, a charitable organisation that provides mental health support to young people aged 5-25 in Merseyside) website, in their support hub, and from January 2024, through 3 schools within the Liverpool Learning Partnership. Project Care UK was signposted as an additional resource by two UK wide organisations that offer free mental health support to adolescents; Kooth commenced signposting in February 2024, and Shout 85258 commenced signposting in March 2024.* |
| Survey administration | Web/E-mail | Methods Procedure *- Project Care UK was fully online, anonymous, open survey hosted on Qualtrics (which specialises in distribution of online surveys),and could be completed in any location and on any internet-connected device.* |
|  | Context | Methods Procedure *- Project Care UK was fully online, anonymous, open survey hosted on Qualtrics (which specialises in distribution of online surveys),and could be completed in any location and on any internet-connected device.* |
|  | Mandatory/voluntary | Methods Procedure *- Participants volunteered to participate by clicking on an anonymous link or scanned the QR code in study adverts.* |
|  | Incentives | Methods Procedure *- In addition, participants were offered the opportunity to enter a prize draw for a £50 Amazon voucher.* |
|  | Time/Date | Methods Recruitment *- Recruitment spanned June 2023 to July 2024.* |
|  | Randomization of items or questionnaires | Methods Procedure *- All measures were presented in a set order (i.e. questions were not randomised).* |
|  | Adaptive questioning | Methods Procedure *- All survey questions were presented in a non-mandatory format, meaning participants were not required to respond in order to continue with the survey.* |
|  | Number of Items | Methods Procedure *- The minimum number of questions that participants completed (e.g., 16-18 year olds) was 136 across 68 pages, while the maximum number of questions that participants completed (e.g., 13-15 year old who failed to complete the GC MCQs twice and provided parental consent) was 153 questions across 74 pages.* |
|  | Number of screens (pages) | Methods Procedure *- The minimum number of questions that participants completed (e.g., 16-18 year olds) was 136 across 68 pages, while the maximum number of questions that participants completed (e.g., 13-15 year old who failed to complete the GC MCQs twice and provided parental consent) was 153 questions across 74 pages.* |
|  | Completeness check | Methods Statistical Analysis *- Completeness checks, which defined participant flow, were conducted following the submission of each questionnaire.* |
|  | Review step | Methods Procedure *- Participants did not have the option to go back and review their answers.* |
|  | Unique site visitor | *N/A* |
|  | View rate (Ratio of unique sur- vey visitors/unique site visitors) | Methods Statistical Analysis *- No statistics on view rate were collected.* |
|  | Participation rate (Ratio of unique visitors who agreed  to participate/unique first survey page visitors) | Methods Statistical Analysis *- No statistics on view rate were collected.* |
|  | Completion rate (Ratio of users who finished the  survey/users who agreed to participate) | Methods Statistical Analysis *- Completeness checks, which defined participant flow, were conducted following the submission of each questionnaire.* |
| Preventing multiple entries from the same individual | Cookies used | Methods Procedure *- Additional measures of fraud detection were also incorporated into the study, including a built in RECAPTCHA score, and function to prevent multiple submissions, though no cookies or IP address checkers were used. These were used to determine eligibility at the data cleaning stage.* |
|  | IP check | Methods Procedure *- Additional measures of fraud detection were also incorporated into the study, including a built in RECAPTCHA score, and function to prevent multiple submissions, though no cookies or IP address checkers were used. These were used to determine eligibility at the data cleaning stage.* |
|  | Log file analysis | *N/A* |
|  | Registration | *N/A* |
| Analysis | Handling of incomplete questionnaires | Methods Statistical Analysis *- Using the MissMech package in R [53], a non-parametric test for Missing Completely At Random (MCAR) indicated that the missingness mechanism was not related to either observed or unobserved variables (P=0.28). This supported the use of complete case analysis (CCA) for the primary models, which included only participants with both pre- and post-intervention data. To assess robustness, a sensitivity analysis was conducted using an intention-to-treat (ITT) approach, where missing post-intervention data were imputed using the mice package in R [54].* |
|  | Questionnaires submitted with an atypical timestamp | ***N/A*** |
|  | Statistical correction | *N/A* |
