# Supplementary material for: Investigating Project Care UK, a Web-Based Self-Help Single-Session Intervention for Youth Mental Health: Program Evaluation
Source: JMIR Ment Health. 2025 Jun 18;12:e72077. doi: 10.2196/72077 (PMC12223457; doi:10.2196/72077)
Supplement: Multimedia Appendix 2 [file mental_v12i1e72077_app2.pdf]

I would seek help from another not listed above (please state).

SH-SSI

Welcome to THE TEENAGE GOALS PROJECT!

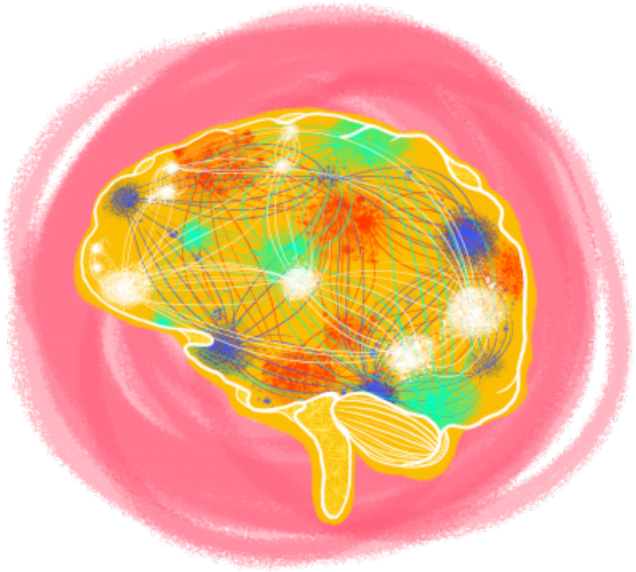

If you have them, please put on your headphones.

The following section will be read-aloud. You will hear stories from scientists and kids like you.

We Need Your Help!

We are scientists from **Stony Brook University**. We study the **brain** and how teenagers reach their **goals**.

Teenagers often tell us they have *lots* of goals. Many of them wonder: ***How* can I work towards the goals that are important to me?** Teenagers tell us the scientific answer to this question is interesting and helpful. We trust them, so we think this information may be helpful for you, too!

But, **we need your help** explaining the science in a way that makes sense to other teenagers like you.

Please help us and future teenagers by completing this activity carefully

How can you help us today?

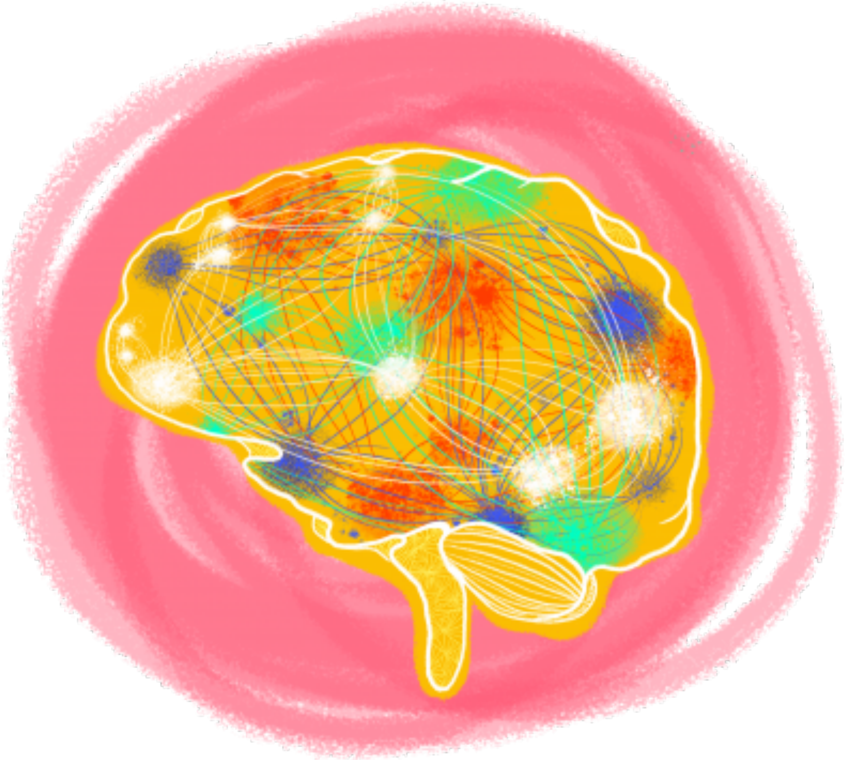

We'd like for you to read what we've written and learn some things science has to say about how the brain works and how we reach our goals. Then, we'd like you to **share your true thoughts and feelings** to help future teenagers.

Remember, the more you share your true thoughts, feelings, and ideas, the more it will **help us learn what to say to future teenagers like you**.

What do teenage goals look like?

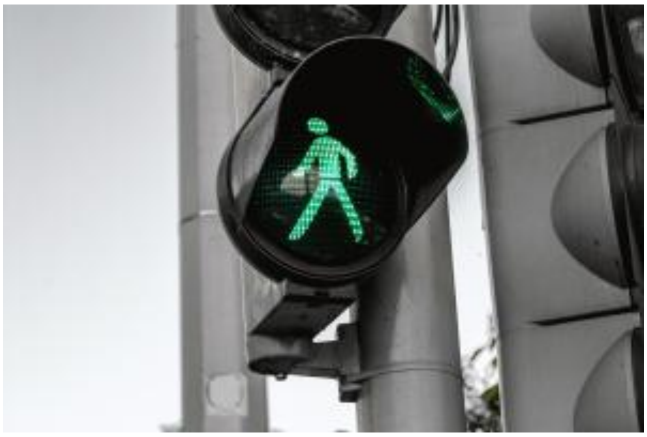

We've talked to teenagers with all kinds of goals -- like building new friendships, doing their best in math class, overcoming a fear, or getting better at dealing with everyday struggles in their lives. **Goals can be lots of things!**

Before we get started, **let's make sure what you learn today relates to *your* life and goals**.

Take a couple of minutes to think of a **personal goal** that really matters to you.
Once you have a goal in mind, describe it in the box below (2 - 5 words).

We don't care about spelling, grammar, or anything like that

Brains are Amazing!

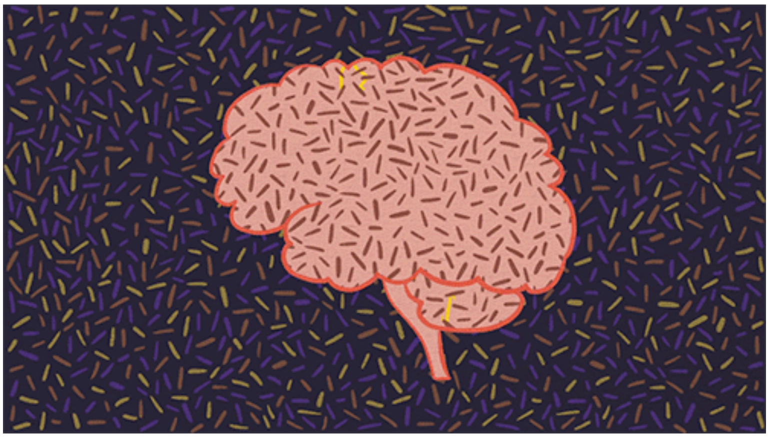

To reach **goals** like the one you just described, people use many different strategies. Different **parts of the brain 'light up'** when we use these different strategies.

Neurons are the **building blocks** of our brains, and they help things 'light up'! They take in *tons* of information from inside and outside our bodies. Then, they send signals to each other to try and make sense of

what's going on.

Brains are Amazing... But They Do Make Mistakes

Our brains are very clever and do an amazing amount of work. But **they can sometimes trick us... without us even realizing it!**

You may be wondering: How can my brain trick me?! Is it tricking me right now??

Luckily, our brains usually aren't tricking us. Our brains often help us make choices that make our lives easier or more fun... like when you see your favorite dessert, and your brain tells you to eat it.

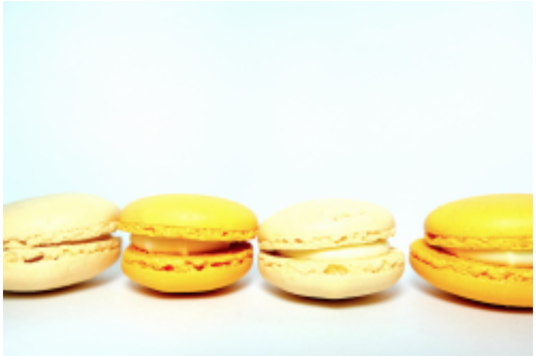

But our brains can make mistakes or jump to wrong conclusions, too.

What part of the brain (sometimes) makes us jump to wrong conclusions?

One really important part of the brain that helps us make choices is the "**Limbic System**". The Limbic System is in the middle area of our brain, and it's all about doing things **really, really fast**.

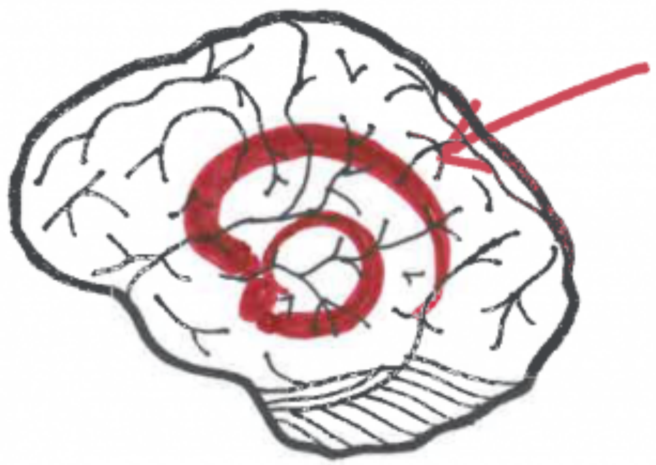

Because this part of the brain has to be so fast, all it can do is look for patterns. We'll be calling it the **pattern-matching part of the brain**

from now on!

Sometimes, looking for patterns can be helpful... like when we see a weird-looking shadow, and our brain tells us to *RUN*, because it might be dangerous.

The **pattern-matching part of the brain** can quickly see the danger and tell us how to react. This is REALLY helpful when there is actual danger around the corner. In that case, the pattern-matching part of our brain is helping us stay safe!

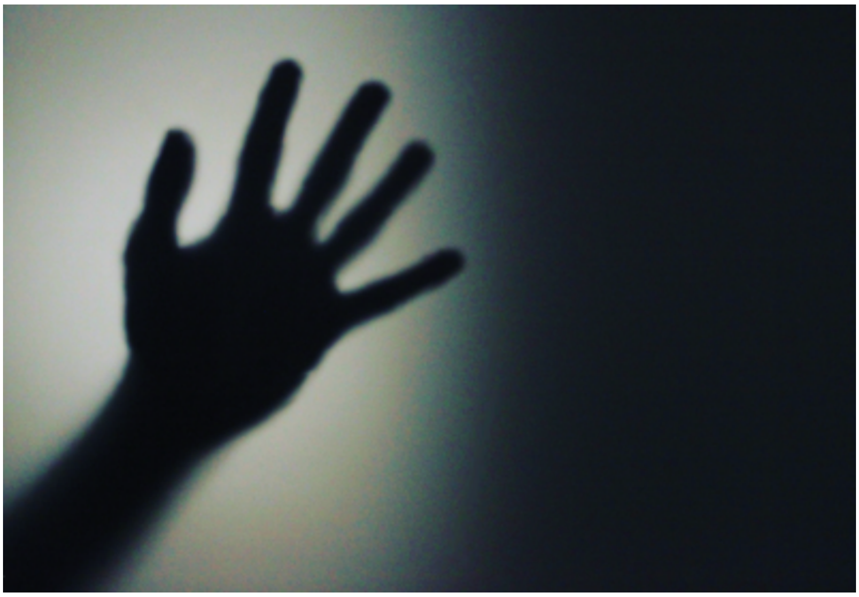

But sometimes, **this pattern-matching part of our brain *can see patterns that aren't really there!*** So, if we **only** listen to this part of the brain, we might jump to the wrong conclusion. Like when we end up running from the shadow... of what was actually a cute kitten.

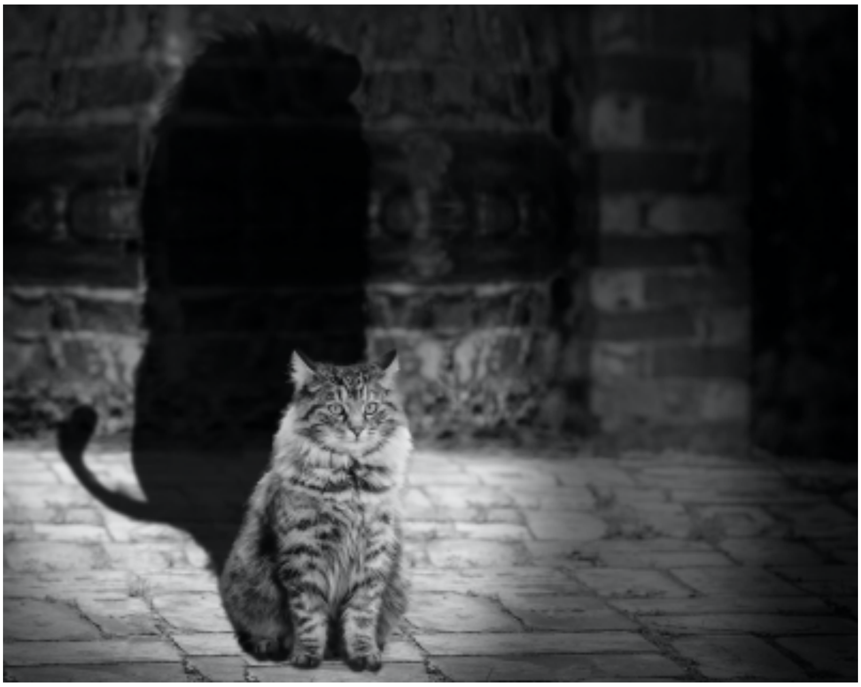

Relying on the pattern-matching part of our brain can mess us up when we're trying to make important decisions

When we think about how to reach our goals (like building friendships, overcoming a fear, or your goal from earlier: ${q://QID128/ChoiceTextEntryValue}), the pattern-matching part of our brains sometimes **jumps to the wrong conclusion** and tells us to do things that seemed to help us before—**even if these things won't**

actually help us with the problem we're facing now.

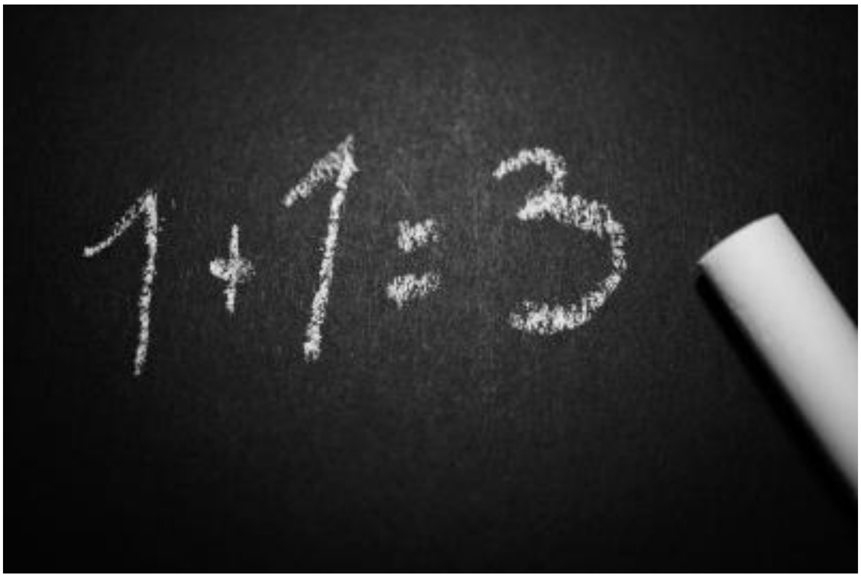

Learning when this part of our brain is jumping to the wrong conclusion can help us have more fun, feel better about ourselves, and reach our goals more often!

One teenager we talked with has a **real-life example** of the **pattern-matching part of the brain** choosing an unhelpful strategy:

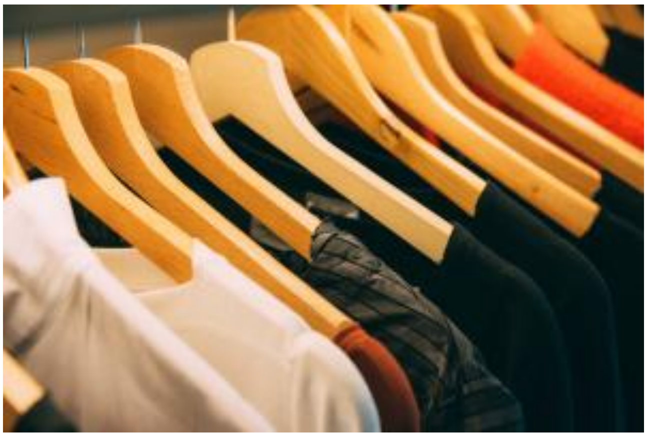

"I had three tests in two days, and by chance I wore jerseys for my favorite team on both days. I got way better grades on those tests than I had earlier in the year. Somehow, the jerseys felt lucky. Soon it felt weird when I didn't wear their jerseys on test days.

A few weeks later, I accidentally wore a regular shirt to a test. I felt bad that day... like I had messed up somehow, or jinxed myself. I still did well on the test, and looking back I know wearing the jerseys wasn't actually helping me get better grades. My brain kept telling me there was a connection between wearing jerseys and getting a good grade. **That part of my brain was wrong**, **but it felt so real at the time.**"

– PB, age 15

Other teenagers, like AM, have had similar things happen:

"I felt like I always had to be super hard on myself to make friends. I would beat myself up for how I said things, what I didn't say, the faces I made, what I wore... everything! People seemed to like me, and I really wanted to keep those friends. I was mean to myself a lot, and it started feeling like beating myself up was the *reason* they stayed friends with me at all. After a while, I started to not like who I was.

Learning why my brain was telling me I needed to be so hard on myself was such a relief. My brain had messed up and matched the pattern of being mean to myself with keeping my friends around. **Even though the part of my brain that looked for patterns was trying to help me, it really just jumped to a wrong conclusion. It was actually making my life way, way harder.**

It didn't happen all at once, but I started giving myself a break when I didn't do everything exactly how I wanted to around my friends. And my friends didn't disappear! If anything, we had a better time together, and I got a chance to like myself more again. **I now know beating myself up wasn't helping me at all. But it's still**

easy to feel like you have to be mean to yourself sometimes. I feel for people who are having a hard time with that now."

– AM, age 16

Like PB and AM learned, when they *automatically* listened to the pattern-matching part of their brains, they ended up doing things that made it harder for them to reach their goals.

It made a **BIG DIFFERENCE** when it came to being **hard on themselves** instead of **kind**.

This is important because... PB and AM's experience happens to **lots of teens.** In fact, lots of teenagers are really, really tough on themselves, or even report **not liking themselves**.

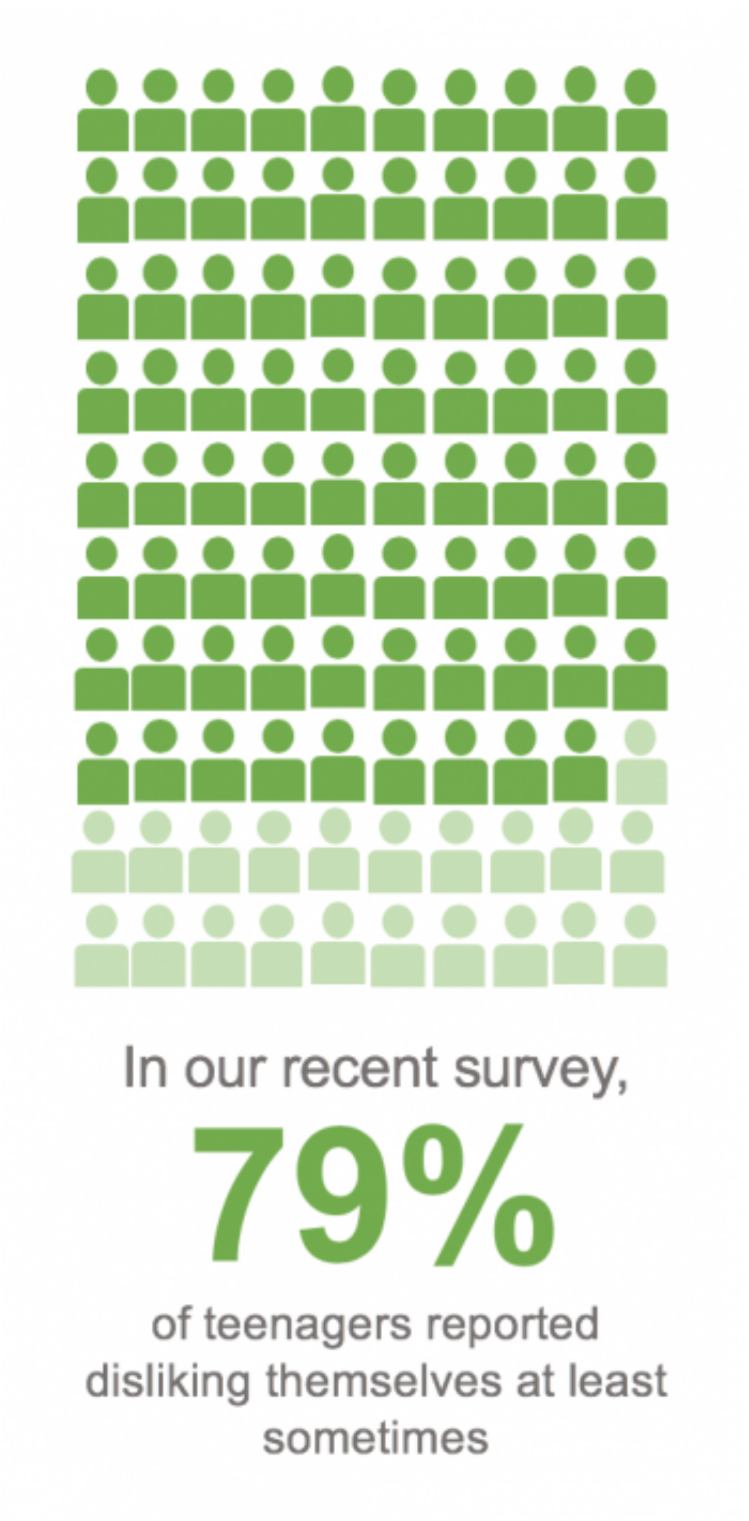
In our recent survey,
79%
of teenagers reported disliking themselves at least sometimes

When we asked teenagers **why they disliked themselves**, most said things like this:

"I have to be really hard on myself to get what I want. When I'm harshest to myself is when I do best."

– ZL, age 12

"Liking myself too much would keep me from getting all the things done that I need to get done. Not liking myself has worked so far, why stop now?"

– JH, age 17

These teenagers' brains **'matched'** reaching their goals with being hard on themselves. So, it makes sense that they decided that being mean to themselves was the only way to succeed.

But here's the problem: **science says the exact opposite**. In one large

study of teenagers, **being meaner to themselves after mistakes made them feel worse about school** over time.

The teenagers who were meaner to themselves had more negative thoughts about school, *and* actually got lower grades overall!

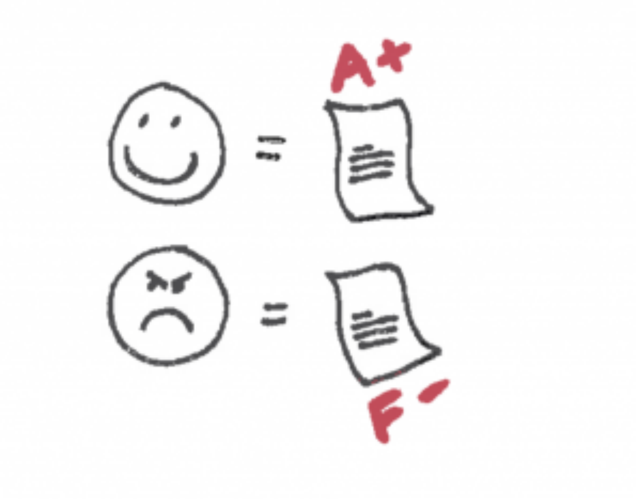

(Source: Cheraghian, *International Journal of Academic Research in Progressive Education and Development*)

It's not just about classes and grades

In another large study, **teenagers who were meaner to themselves following mistakes ended up less happy with their social lives**

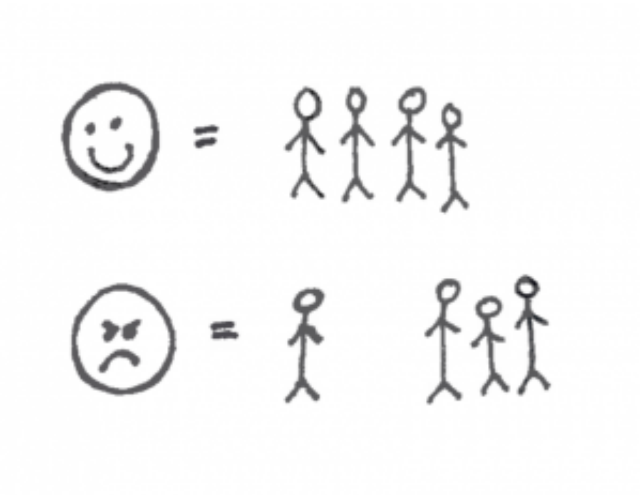

(Source: Terry, *Self and Identity*)

So: Many teenagers we talked to reached goals...even after being mean to themselves.

But it turns out being mean to themselves was making it HARDER, not easier, to reach the goals they cared about

-

In fact, in all of these studies, **teenagers who were kinder to themselves after mistakes were less likely to feel badly about school and more likely to be happy with their social lives**

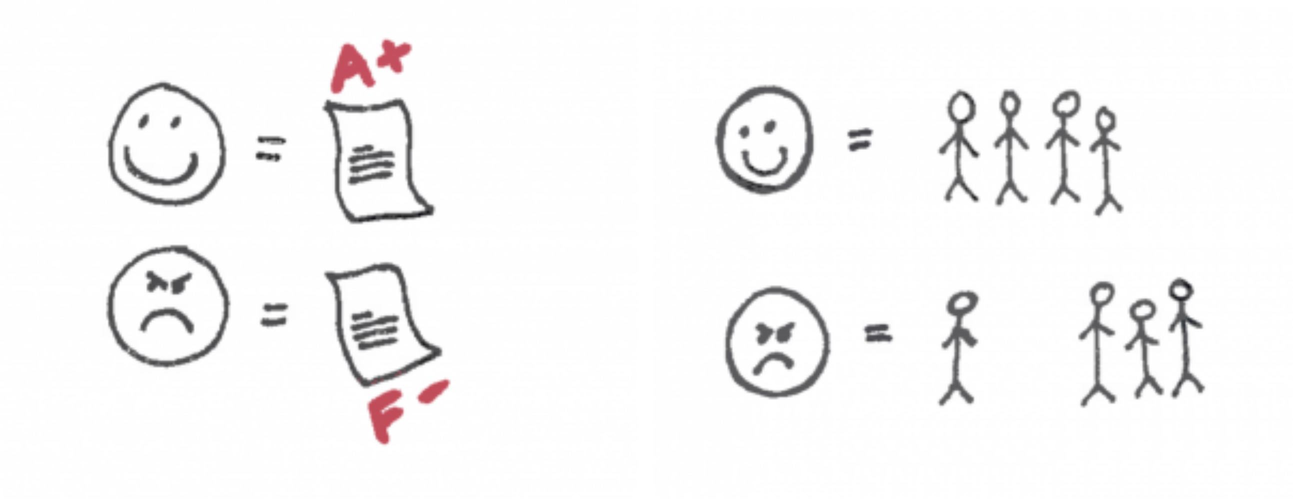

Plus, being kind them themselves seemed to HELP them notice and learn from their mistakes. Teenagers who were kinder to themselves **were better at knowing when they had made a mistake** in an embarrassing social situation.

(Source: Leary, *Journal of Personality and Social Psychology*)

So, science shows **being kind to ourselves**

(instead of mean to ourselves) helps us reach our goals AND helps us figure out how well we're really doing

Why is being kind to ourselves more helpful than being mean to ourselves?

...One study gives us some interesting clues! When people are mean to themselves after a mistake, a certain part of their brain **'lights up'**.

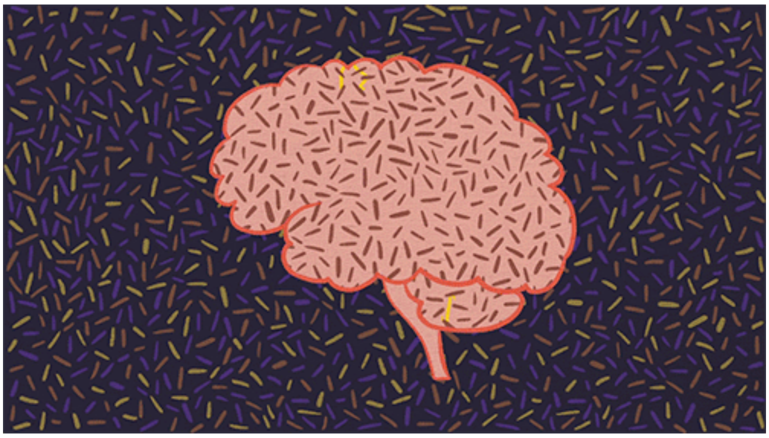

When this part of the brain is **too active**, it stops us from taking action. It gets people stuck in a **negative thinking spiral** instead of **taking action.**

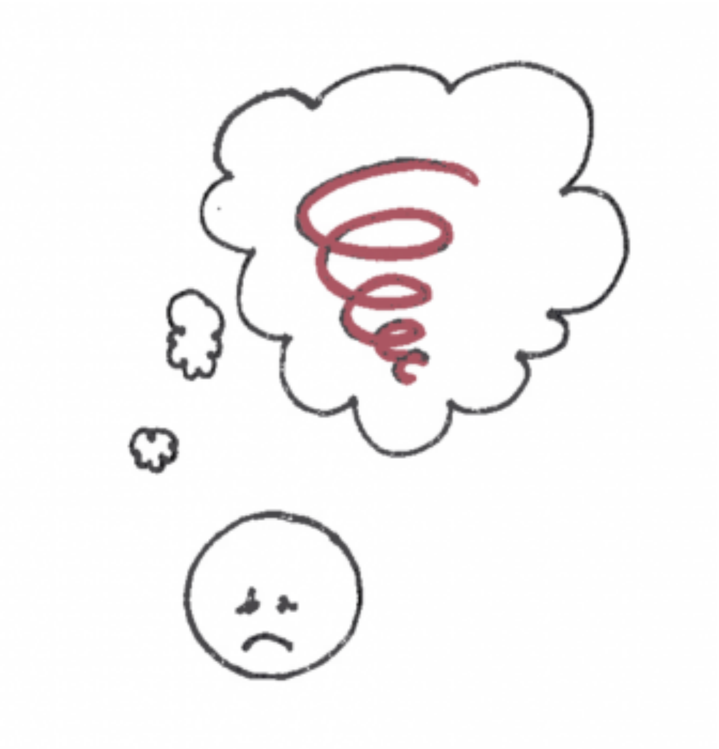

(Source: Longe, Neuroimage)

Sometimes, it's good to think instead of act -- like when we're making really important or difficult choices. But many times, we *have* to take action in order to reach our goals, especially after setbacks (like working out to try out for a sports team, or introducing ourselves to people in order to make new friends).

Teenagers who are kind to themselves after making mistakes find it MUCH EASIER to take action toward making their goals

happen.

...But people who are **mean** to themselves after mistakes may get caught in a negative thinking spiral. This makes is harder to get things done.

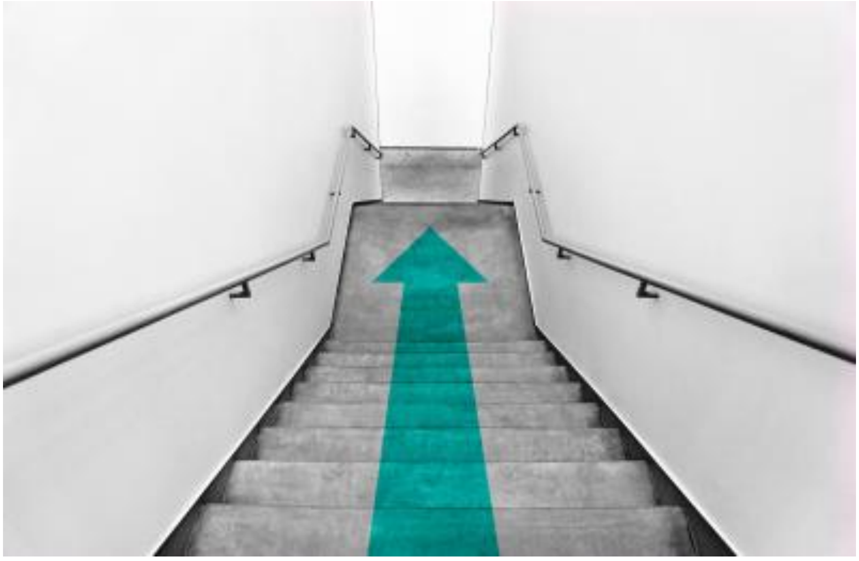

Learning the science behind why this is true can make a difference! We talked with teens about how the **pattern-matching part of the brain** sometimes jumps to wrong conclusions - like, "being hard on myself will help me reach my goals." Afterwards, **a lot of them changed how they responded to making mistakes.**

Remember ZL, who thought they had to be their own harshest critic? After they learned the science they said:

"The part of our brain that looks for patterns can lead us wrong, which is why it's nice to learn actual science about what works. I'm still not sure if I deserve kindness from myself, but I'm now willing to try."

And JH, who thought they needed to dislike themselves to succeed? After they learned the science they said:

"Wow, I feel a little silly for being so sure I had to be so mean to myself. It's good to know I wasn't alone in thinking that. But it's also good to know I can be kinder to myself and still do everything I want to do, no matter what the pattern-matching part of my brain tells me."

ZL and JH aren't alone. May teenagers are still hard on themselves. But our surveys say this is changing: **more and more teenagers** are starting to see being kind to themselves is the better way to go

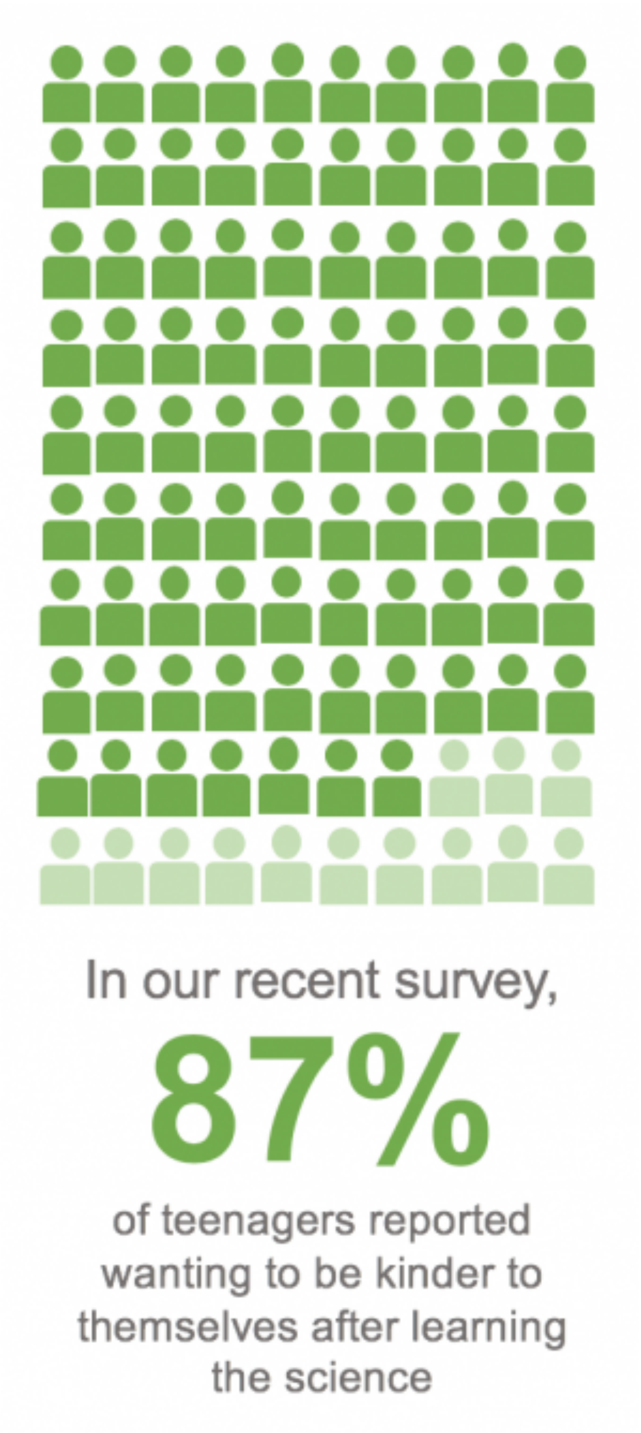

In fact, these teenagers helped us come up with a new way to describe choosing to be mean to ourselves after making a mistake:

Not being kind to ourselves when we're having a hard time, or after we've made a mistake, is like trying to grow a plant in a dark room

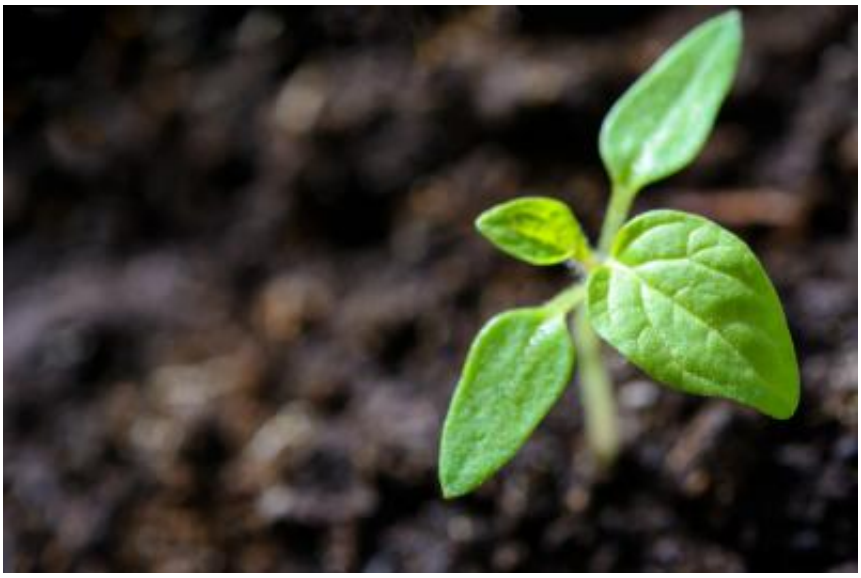

It might *seem* like a dark, quiet, safe room should help a plant grow (you're protecting the plant from bad weather and hungry critters!), just like it may *seem* like being mean to yourself should help you achieve your goals.

But actually, it's super hard to grow a plant in a dark space -- **the plant won't have the sunlight it needs to actually grow**

And like we learned earlier, **being mean to ourselves makes it harder, not easier, to reach our goals**

So, being mean to ourselves (*putting a plant in a dark room*) keeps us from getting what we need (*direct sunlight*) and ends up making it harder to take action and reach our goals

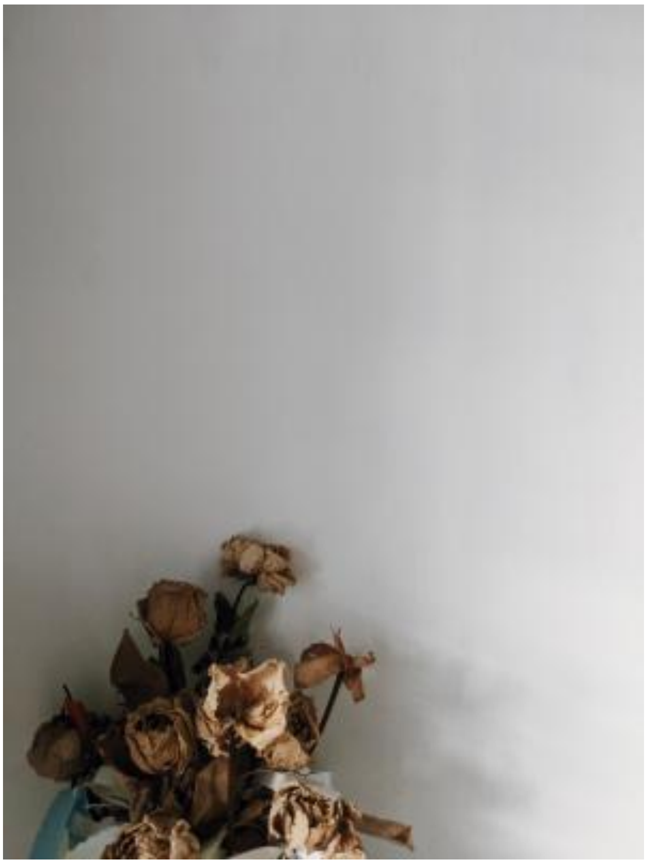

We need your help to explain this to other teenagers

Think about a time when someone you know tried reach a goal (*make a plant grow*) but was mean to themselves after a mistake (*put their plant in a dark room*).

Use the plant + sunlight comparison however you'd like to explain how this person might have more easily reached their goal if they were KIND to themselves instead. Feel free to talk about the science on being kind to ourselves vs. being mean to ourselves, and we don't care about spelling, grammar, or anything like that.

Please use about 2-3 sentences

Let's review what we've covered so far:

- Sometimes, the pattern-matching part of our brain moves too quickly and makes mistakes

- As a result, our brain can tell us to solve problems in ways that aren't

super helpful

- This process can lead some teenagers to believe they should be mean to themselves, instead of kind, after failures

- But! **Science shows being kind to ourselves (and not being mean to ourselves) is more likely to make us feel better and succeed**

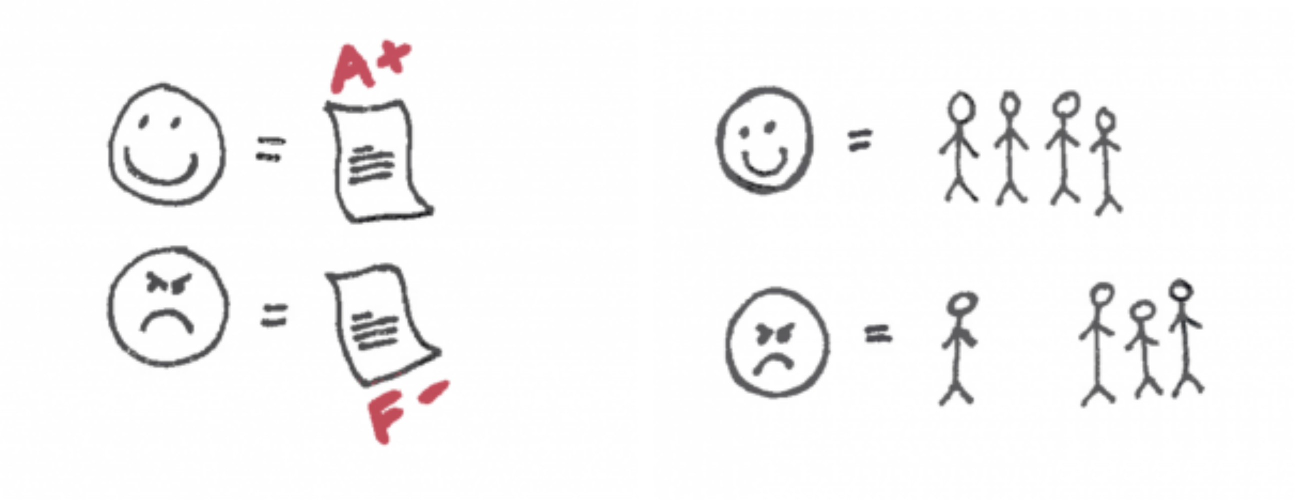

Still, for many teenagers, being kind to themselves feels weird or difficult at first.

Here are 3 of the biggest obstacles teenagers have mentioned about being kind to themselves:

"What if you don't always think you deserve kindness from yourself?"
"What if I try to be kind to myself, and I'm bad at it, or it feels fake?"
"What if I want to be kind to myself, but don't remember to do it when things are hard?"

Luckily, **science can help us face these obstacles**

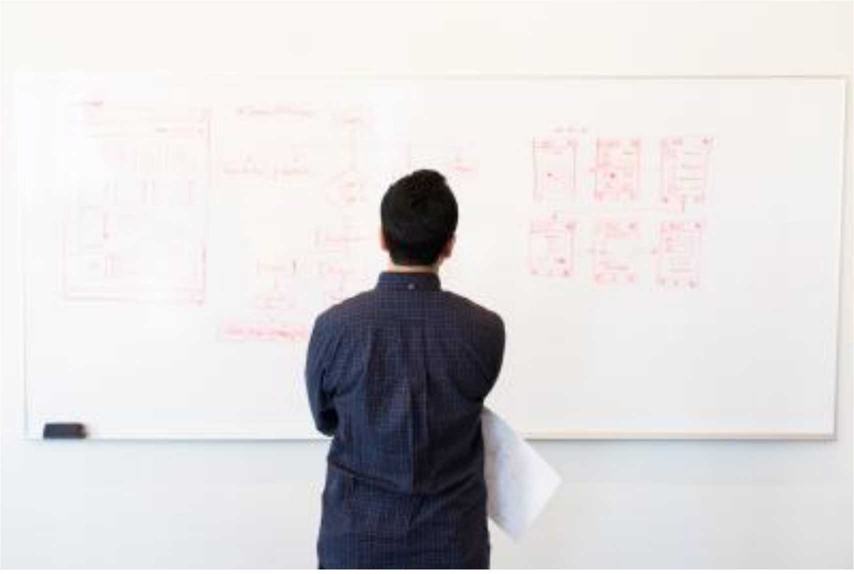

Obstacle 1: What if you don't think you always deserve kindness from yourself?

First, that's a tough spot to be in at any point, and we're sorry if that's where you're at. Luckily, science can help us figure out what to do next.

If we **change what we *do*** (for example, learning the guitar), science

shows it can **change how we *think*** (like thinking, "I could get good at guitar!") and **change how we *feel*** (like feeling proud of your growing guitar skills).

By changing what we ***do***, what we *think* can 'catch up' to how we act more and more over time.

This means: **by acting nice to ourselves a little each day, we can end up *thinking* more and more that we deserve self-kindness, and *feeling* better over time.**

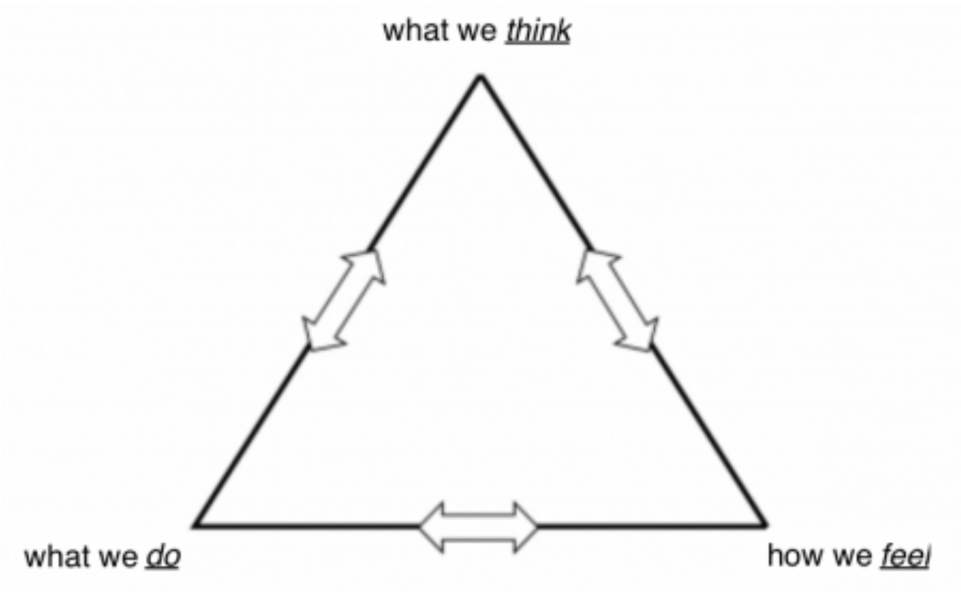

(Source: Beck, Behavior Therapy)

We're not pretending it will be easy, but ZL from earlier also didn't think they deserved kindness from themselves. After a few weeks, they told us this:

"I'll be honest, I didn't believe any of it at first. Thinking I didn't deserve kindness sucked. But I'd thought that way for a long time... so even if I wanted to change things, I felt like there was no way that I actually could. But I finally gave "acting kind" a shot -- after a while, I started noticing my thoughts were a little less harsh toward myself, and being kind to myself started getting easier. I'm not all the way there yet or anything, but it's unreal how much better my life has already changed for the better."

Obstacle 2: What if you try to be kind to yourself, and you feel like you're bad at it, or it feels fake?

That's a super common thing to feel! For people who haven't practiced being kind to themselves before, it can feel weird at first.

Science has shown that being kind to yourself is like a muscle. People who practice it get better at it over time, even if they felt like

they weren't good at it at the beginning (Source: Neff, *Self and Identity*).

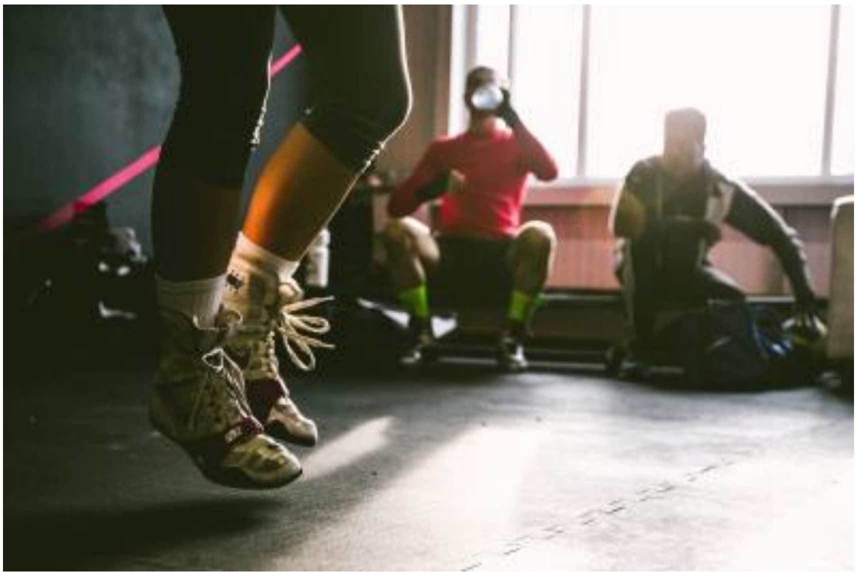

Obstacle 2: What if you try to be kind to yourself and you feel like you're bad at it, or it feels fake?

Teenagers we've talked to have helped us find a good **starting point** for being kinder to themselves: **treating themselves like they would normally treat a good friend** who was having a hard time.

We can all think of examples of how we would try to treat a good friend, and science shows thinking about how we'd help others makes it easier to be kind to ourselves (Source: Breines, Journal of Experimental

& Social Psychology)

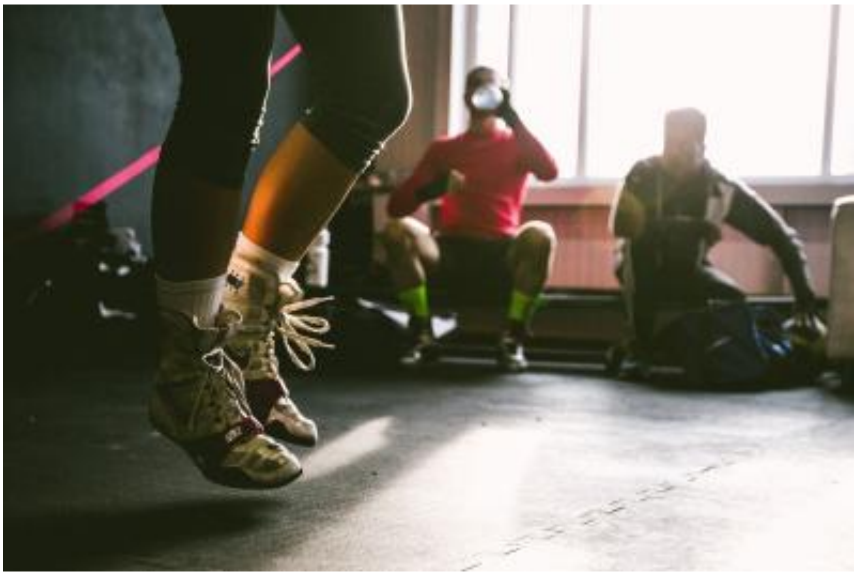

It won't happen overnight for everyone, but when we followed up with JH, who we met earlier, after a couple of weeks they said:

"Is it weird to feel like you don't know how to be nice to yourself? Because that's how I felt for the first bit. The good thing is, I kept telling myself it was similar to being nice and supportive to a friend, which I think I'm pretty good at. That made it a little easier. I don't know if I'm 'good' at being kind to myself compared to other people, but whatever! What I'm doing now is working much better for me than what I was doing before."

Obstacle 3: What if you want to be kind to yourself but have a hard time remembering to do it when things are hard?

Many teenagers also said sometimes they wanted to be kinder to themselves, but they forget to actually do it in the moment.

Luckily, our bodies sometimes know when we're needing a little extra kindness before we even realize it!

Science shows that physical feelings you associate with feeling down (like stomachaches, headaches, or tight shoulders) can be a sign to start being kind to yourself. For some people, uncomfortable feelings in our body are easier to spot than difficult thoughts or emotions.

You're the expert on yourself, so you can choose which signs may help you know when to use kindness!

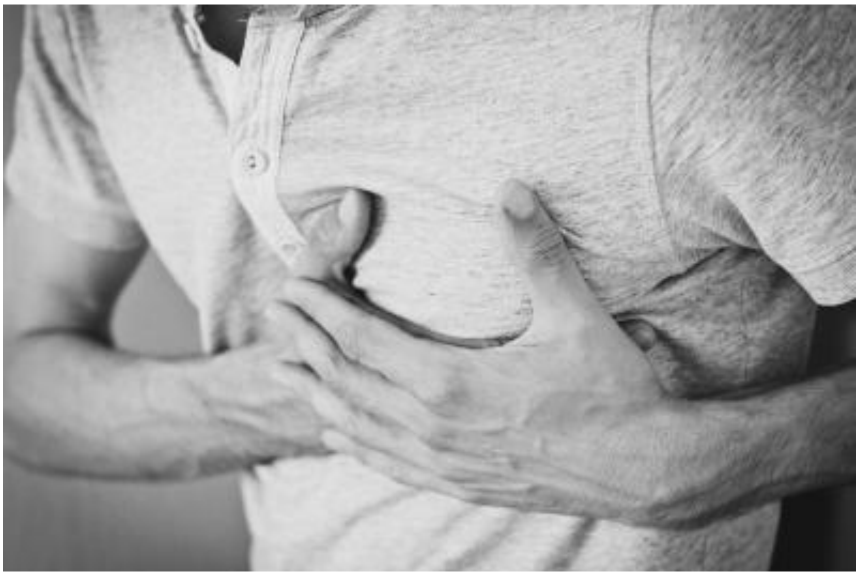

(Source: Harvey, *Perspectives on Psychological Science*)

Want another real life example? MD took one of our surveys, and they said:

"I wasn't really sure how I would remember to actually be nice to myself when things were actually hard. It was nice to hear other people didn't always remember to do it either, so I didn't need to get it right every single time in order for it to work.

I also really liked the idea of using something physical to remind me to be kind to myself. Sometimes, my thoughts are hard to keep track of, but I know if my chest

feels heavy I've probably been mean to myself. Now, just being nice to myself about some little things each day makes me feel better overall."

Let's make this more specific to you and your life. Please choose which of these options (you can select as many as you want!) might make it hardest for you to be kind to yourself in your day to day life.

- ☐ At least sometimes I don't think I deserve kindness from myself
- ☐ What if I try to be kind to myself and I'm bad at it?
- ☐ What if I want to be kind to myself but have a hard time remembering to do it when things are hard?

Please tell us in 2-3 sentences about **how the science you've learned could help overcome these obstacles**: ${q://QID76/ChoiceGroup/SelectedChoices}

Spelling, grammar, and anything like that don't matter to us, we just

want to hear what you've learned about the science

Alright, let's review for a second:

- Being mean to ourselves when we make mistakes is like trying to grow a plant in a dark room

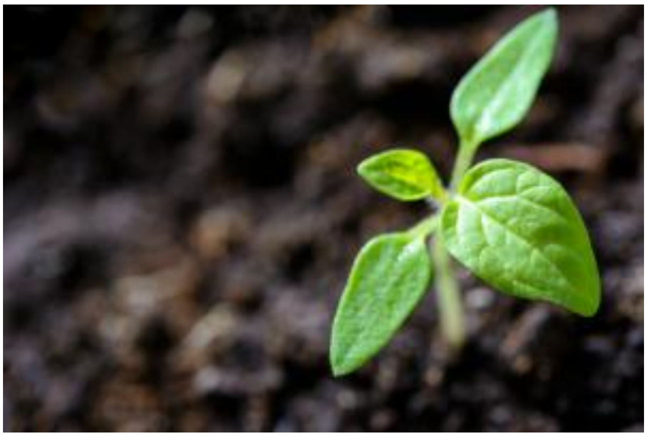

- There are common obstacles to being kind to ourselves. (And knowing which obstacles might be harder for you is a good first step to

you beating them!)

- While knowing ***how*** to start can be hard, **we can treat ourselves like we would treat a friend**

- While recognizing ***when*** to use kindness can be hard, **paying attention to feelings in our body can help**

Let's practice using what we've learned to help others and ourselves

Please tell us in 2-3 sentences about a recent time when you felt like you were really mean to yourself following a mistake. This could be related to friends, family, school, work, or something else.

To keep your information safe, please do not include your full name or any other person's full name

Now imagine that the same event happened to another teenager just like you. **What could you say to help them understand that being kind to themselves will be helpful to them?**

Please give them 1-2 paragraphs of advice. They will appreciate hearing your own thoughts, feelings, and experiences + some of the science you've learned

Thanks so much for helping out future teenagers!

Program Feedback Scale

Thank you for completing our activity!

You're helping us understand teens' experiences so
