# Supplementary material for: Investigating Project Care UK, a Web-Based Self-Help Single-Session Intervention for Youth Mental Health: Program Evaluation
Source: JMIR Ment Health. 2025 Jun 18;12:e72077. doi: 10.2196/72077 (PMC12223457; doi:10.2196/72077)
Supplement: Multimedia Appendix 3 [file mental_v12i1e72077_app3.docx]

Online Supplementary Materials S1. Gillick competence assessment to allow 13-15 year olds to self-consent

All young people completed the GC MCQs post consent, which entailed 4 multiple choice questions, each of which was related to one aspect of GC (e.g., purpose, process, benefits, harms). The questions were drafted by Maria Loades, with input from the University of Bath Psychology Ethics Committee, 2 undergraduate psychology students at Bath, and the LAMP YPAG. A full breakdown of the questions used can be found in Loades et al. (2024). During the first 6 months of recruitment, the questions were not used to directly measure GC, rather to explore the feasibility of this process within online research. In January 2024, a third branch was added. Young people aged 13-15 could opt to show GC or provide parental consent, thus branching logic was used to display the appropriate pathway. Those who opted to complete the GC MCQs were shown the information sheet, and the GC MCQs. Young people who failed to answer all MCQs correctly were displayed an ineligibility message and offered the opportunity to attempt to show GC again or provide parental consent. If young people failed to answer all MCQs correctly again they were required to provide parental consent. If young people answered all MCQs correctly on either attempt, they moved on to pre-intervention measures.
