# Supplementary material for: Investigating Project Care UK, a Web-Based Self-Help Single-Session Intervention for Youth Mental Health: Program Evaluation
Source: JMIR Ment Health. 2025 Jun 18;12:e72077. doi: 10.2196/72077 (PMC12223457; doi:10.2196/72077)
Supplement: Multimedia Appendix 4 [file mental_v12i1e72077_app4.docx]

## **Multimedia Appendix 4. Preliminary efficacy—results of preregistered data analysis**

### **Important Note**

The following results reflect analyses conducted in accordance with the original pre-registered data analysis plan listed on the Open Science Framework (OSF). To account for sample size and data completeness, we later revised the plan and uploaded the updated version to OSF. While the main manuscript followed this revised plan, for transparency, we opted to also present the results of the original data analysis plan. To avoid redundancy between the main manuscript and Multimedia Appendix 3, table content corresponding to preintervention assessment mean (SD), postintervention assessment mean (SD), β (SE), Cohen *d* (95% CI), and improved case data (n, %) are omitted.

### **Preliminary Efficacy**

Of the 813 participants, 329 (40.5%) initiated the post-intervention assessment survey. Statistically significant improvements were observed for three of the outcome measures, including increased hope (n=326/813, 40.1%, *P*<.001), decreased hopelessness (n=309/813, 38%, *P*<.001), and decreased negative beliefs about self-compassion (n=300/813, 36.9%, *P*<.001). Help-seeking tendencies did not exhibit any significant change (n=298/813, 36.7%, *P*=.13). Detailed pre-post intervention comparisons are presented in Table 3.1.

Table 3.1. Pre-post intervention comparisons of hope, hopelessness, self-compassion, and help-seeking. ^a^

|  | *t* test (*df*) | *P* value |
| --- | --- | --- |
| SHS pathways subscale^b^ | -13.07 (325) | <.001 |
| BHS^c^ | 12.92 (308) | <.001 |
| BSCS^d^ | 11.12 (299) | <.001 |
| GHSQ^e^ | -1.54 (297) | .13* |

^a^Pre and postintervention comparisons for hope (State Hope Scale pathways subscale), hopelessness (Beck Hopelessness Scale), beliefs about self-compassion (Beliefs About Self Compassion Scale), and help-seeking (General Help-Seeking Questionnaire). Values for *t* tests, *dfs*, and *P* values are reported.

^b^SHS: State Hope Scale.

^c^BHS: Beck Hopelessness Scale.

^d^BSCS: Beliefs About Self-Compassion Scale.

^e^GHSQ: General Help-Seeking Questionnaire
