# Supplementary material for: Investigating Project Care UK, a Web-Based Self-Help Single-Session Intervention for Youth Mental Health: Program Evaluation
Source: JMIR Ment Health. 2025 Jun 18;12:e72077. doi: 10.2196/72077 (PMC12223457; doi:10.2196/72077)
Supplement: Multimedia Appendix 5 [file mental_v12i1e72077_app5.docx]

## **Multimedia Appendix 5. Preliminary efficacy—sensitivity analysis**

Table 4.1. Pre-post intervention comparisons of hope, hopelessness, self-compassion, and help-seeking^a^

|  | Preintervention assessment, mean (SD) | Postintervention assessment, mean (SD) | β (SE) | *t* test (*df*) | *P* value | Cohen *d* (95% CI) |
| --- | --- | --- | --- | --- | --- | --- |
| SHS pathways subscale^b^ | 11.79 (4.71) | 14.63 (5.14) | 2.85  (0.27) | 10.40 (1906.4) | <.001 | 0.75^c^  (0.66, 0.83) |
| BHS^d^ | 2.65 (1.07) | 2.08 (1.13) | -0.57  (0.06) | -9.00 (1543.5) | <.001 | -0.76^c^  (-0.92, -0.61) |
| BSCS^e^ | 3.22 (0.80) | 2.63 (0.91) | -0.58  (0.05) | -10.12 (2717.7) | <.001 | -0.64^c^  (-0.78, -0.50) |
| GHSQ^f^ | 3.28 (0.78) | 3.33 (0.87) | 0.05  (0.04) | 1.30 (2325.1) | .19 | 0.09^c^  (-0.04, 0.21) |

^a^Values are based on an intention-to-treat sample with missing post-intervention values imputed using multiple imputation. Analyses reflect pooled estimates across imputations following Rubin’s Rules. Pre and postintervention comparisons for hope (State Hope Scale pathways subscale), hopelessness (Beck Hopelessness Scale), beliefs about self-compassion (Beliefs About Self Compassion Scale), and help-seeking (General Help-Seeking Questionnaire). Values represent number of cases (n), pooled means, pooled SDs, pooled fixed effect estimates (β), *dfs*, *t* test values, *P* values, and pooled effect sizes (Cohen *d*) with 95% CI. Effect sizes are classified as small, medium, or large. Pre-post changes in hope, hopelessness, and beliefs about self-compassion were significant.

^b^SHS: State Hope Scale.

^c^Medium effect size.

^d^BHS: Beck Hopelessness Scale.

^e^BSCS: Beliefs About Self-Compassion Scale.

^f^GHSQ: General Help-Seeking Questionnaire.
